# Powerful and accurate case-control analysis of spatial molecular data with deep learning-defined tissue microniches

**DOI:** 10.1101/2025.02.07.637149

**Published:** 2025-02-08

**Authors:** Yakir Reshef, Lakshay Sood, Michelle Curtis, Laurie Rumker, Daniel J. Stein, Mukta G. Palshikar, Saba Nayar, Andrew Filer, Anna Helena Jonsson, Ilya Korsunsky, Soumya Raychaudhuri

**Affiliations:** 1 Center for Data Sciences, Brigham and Women’s Hospital, Boston, MA, USA; 2 Division of Genetics, Department of Medicine, Brigham and Women’s Hospital and Harvard Medical School, Boston, MA, USA; 3 Department of Biomedical Informatics, Harvard Medical School, Boston, MA, USA; 4 Broad Institute of MIT and Harvard, Cambridge, MA, USA; 5 Division of Rheumatology, Inflammation, and Immunity, Department of Medicine, Brigham and Women’s Hospital and Harvard Medical School, Boston, MA, USA; 6 NIHR Birmingham Biomedical Research Centre, University Hospitals Birmingham NHS Foundation Trust and Department of Inflammation and Ageing, College of Medicine & Health, University of Birmingham, Birmingham, UK; 7 Birmingham Tissue Analytics, College of Medicine and Health, University of Birmingham, Birmingham, UK; 8 University of Colorado Anschutz Medical Campus, Division of Rheumatology, Aurora, CO, USA

## Abstract

As spatial molecular data grow in scope and resolution, there is a pressing need to identify key spatial structures associated with disease. Current approaches often rely on hand-crafted features such as local abundances of manually annotated, discrete cell types, which may overlook important signals. Here we introduce variational inference-based microniche analysis (VIMA), a method that combines deep learning with principled statistics to discover associated spatial features with greater flexibility and precision. VIMA uses a variational autoencoder to extract numerical “fingerprints” from small tissue patches that capture their biological content. It uses these fingerprints to define a large number of “microniches” – small, potentially overlapping groups of tissue patches with highly similar biology that span multiple samples. It then uses rigorous statistics to identify microniches whose abundance correlates with case-control status. We show in simulations that VIMA is well calibrated and more powerful and accurate than other approaches. We then apply VIMA to a 140-gene spatial transcriptomics dataset in Alzheimer’s dementia, a 54-marker CO-Detection by indEXing (CODEX) dataset in ulcerative colitis (UC), and a 7-marker immunohistochemistry dataset in rheumatoid arthritis (RA), in each case recapitulating known biology and identifying novel spatial features of disease.

## Main

Spatial profiling of tissue samples has been a foundational tool for diagnosing and understanding disease starting from the discovery of red blood cells and capillaries in the 1600s with the microscope^1^. Since the introduction of haematoxylin and eosin (H&E) staining in the late 1800s^2^, spatial *molecular* techniques have provided increasing insight into tissue biology and are now a pillar of both clinical medicine and biomedical research^3–6^. In recent years, the scale and richness of spatial molecular modalities have dramatically increased. Highly multiplexed protein modalities such as imaging mass cytometry^7^ and CODEX^8^ have enabled the spatial measurement of scores of proteins, and transcript-based modalities such as MERFISH^9^, Xenium^10^, and others^11–14^ can now profile thousands of genes at high spatial resolution.

However, the very richness of modern spatial data has made mechanistic research challenging. Historically, spatial data have been analyzed through manual inspection and the development of highly detailed, bespoke assessment systems. For example, in rheumatoid arthritis, synovial inflammation is still graded using a complex scoring system that relies on qualitative assessments made by a pathologist^15^, and tumors are characterized using assessments of cancer cell morphology and of lymphocyte arrangements and proportions in specific tumor microenvironments^16,17^. These assessment systems limit discoveries to those that fit the paradigms under which they were established and scale poorly as we assay more genes and proteins.

Recently published studies of spatial molecular data have used more structured approaches to define and annotate spatial neighborhoods and then test them for case-control differences^18–21^. These approaches generally use a “local averaging paradigm”, in which a spatial neighborhood is represented via aggregate properties such as cell-type abundances or average expression profiles. This paradigm ignores fine-grained spatial relationships among cells as well as any non-cellular structures that may be relevant. Sophisticated methods, including deep learning-based methods and methods outside the local averaging paradigm, have been developed to improve our ability to identify spatially varying genes^22^, impute missing genes^23^, characterize tissue architecture^23–27^, and increase spatial resolution^28,29^. But these methods are either designed to examine variation within a single sample rather than variation across multiple samples as is required for case-control analysis, or they are designed for specific technologies such as spot-based technologies. Thus, to date no method for performing case-control analysis on a broad range of spatial modalities has gone beyond the local averaging paradigm.

Here we present VIMA, a method that combines deep learning with principled high-dimensional statistics to rigorously and flexibly conduct association testing in spatial datasets across the full range of spatial modalities. VIMA uses a variational autoencoder^30^ to learn a “fingerprint” for each of many small tissue patches in a spatial dataset. Each fingerprint is a simple numerical vector that describes the key biology of its tissue patch while excluding sample-specific or batch-specific nuisance variation. VIMA then uses these fingerprints to build a nearest-neighbor graph of tissue patches that enables it to define many small, potentially overlapping groups of highly similar tissue patches, which we term *microniches*. Finally, it performs statistical tests to search for associations between any sample-level attribute and the abundances of these microniches across samples. VIMA produces both a global statistical test for the presence of any case-control difference in the spatial data and a local statistical test identifying the set of specific microniches driving that difference at a given false discovery rate. VIMA can be applied to any spatially resolved molecular technology, is well powered even at the modest sample sizes typical of research cohorts, and avoids traditional, parameter-intensive preprocessing steps such as cell segmentation or clustering of cells into discrete cell types.

We apply VIMA to both simulated and real datasets. We first conduct simulations built with real spatial transcriptomic data to which we add synthetic case-control signals and assess VIMA’s power and spatial accuracy. Our simulations show that VIMA is statistically well-calibrated and powerfully and accurately detects a broad range of case-control signals. To understand the value of key features of VIMA such as patch fingerprints and microniches, we also compare it to a series of modified VIMA-like methods that lack these features and find superior power and spatial accuracy compared to these alternatives. Our comparisons include, among others, the local averaging paradigm as instantiated through representation of patches by their average expression profiles.

We then apply VIMA to three datasets generated using MERFISH, CODEX, and immunohistochemistry, respectively^18,31,32^. For each technology, we compare to the local averaging paradigm by representing each patch using average expression profiles as well as cell-type abundances if cell segmentation is available. In each case, VIMA both recapitulates the signals that are detectable within the local averaging paradigm and reveals novel disease-associated spatial features that are not.

## Results

### Overview of methods

VIMA is based on two central concepts: using deep learning to represent each tissue patch in a dataset with a numerical “fingerprint” summarizing its essential biology, and using the fingerprints to define small groups of highly similar tissue patches called “microniches” that form the basic unit of association testing.

VIMA takes as input 1) a multi-sample spatial molecular dataset that profiles protein levels, transcript levels, stain intensities, or some combination of these, and 2) a table of sample-level metadata containing case-control status and any other relevant covariates such as patient age or sex. VIMA’s output consists of 1) a numerical fingerprint for each tissue patch in the dataset that summarizes the contents of that tissue patch and does not depend on case-control labels, 2) a microniche defined for each tissue patch in the dataset that also does not depend on case-control labels, 3) a global P-value indicating the statistical significance of the aggregate spatial differences between case samples and control samples, and 4) a subset of tissue patches whose microniches drive this association, at a specified false discovery rate threshold.

**Figure 1:**
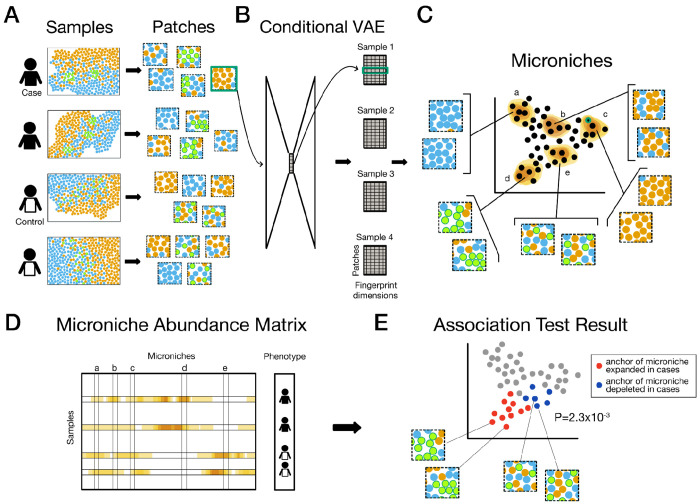
A schematic depiction of VIMA. **A)** VIMA generates a large number of small, square, partially overlapping tissue patches from each sample. **B)** VIMA trains a conditional variational autoencoder on all the tissue patches to learn a low-dimensional vector representation (a “fingerprint”) of each patch that summarizes its biological contents, such that similar patches have similar fingerprints. **C)** VIMA uses the fingerprints to build a nearest-neighbor graph of patches and then defines, for each patch, a microniche anchored at that patch to which each other patch belongs in proportion to how close it is to the anchor patch in the nearest neighbor graph. **D)** VIMA counts the fraction of patches in each sample belonging to each microniche and represents this information as a samples-by-microniches matrix called the microniche abundance matrix, alongside the sample-level phenotype. **E)** VIMA correlates each column of the MAM with the phenotype and uses statistical tools to assess which correlations are significant after accounting for multiple hypothesis testing and correlation among tests. We visualize the result by coloring each patch in the UMAP according to the correlation of the abundance of its respective microniche to the phenotype. Patches anchoring non-significant microniches are colored in gray. The P-value overlaid on the plot is a global P-value that reflects the overall significance of the case-control association.

To create the patch fingerprints, VIMA rasterizes each sample into pixels, performs principal components analysis (PCA) to reduce the number of markers per pixel to a smaller set of “meta-markers”, and integrates these meta-markers using Harmony^33^ to remove non-spatial batch effects and sample-specific artifacts (Methods). VIMA then transforms each sample in the dataset into a collection of partially overlapping, square tissue patches (Fig. 1A) that are 400x400um by default. Depending on cellular density, each tissue patch may contain tens to hundreds of cells. Since the tissue patches overlap, a 1x1cm tissue fragment yields 10,000 tissue patches with meta-marker information on 1 million pixels. VIMA then uses the tissue patches to train a conditional variational autoencoder^34^ with a ResNet18-style architecture^35^ that transforms each tissue patch into a small numerical vector (the “fingerprint”; Fig. 1B); the autoencoder is explicitly designed to remove the effects of sample ID and batch ID on the fingerprint (Methods). Each sample is subsequently represented as a collection of numerical patch fingerprints, enabling the use of standard bioinformatics tools, such as clustering and UMAP construction on the tissue patches if desired.

To define microniches, VIMA builds on our previous work in single-cell analysis^36^. It first constructs a nearest-neighbor graph of the patch fingerprints which can be visualized with UMAP^37^ (Fig. 1C). It then defines a single microniche anchored at each patch *p*: every other patch *p’* belongs to that microniche according to the probability that a random walk in the graph from *p’* will arrive at *p* after *s* steps (Methods and Fig. 1C). VIMA chooses the length *s* of the random walk in a data-dependent manner (Methods).

VIMA uses the microniches to compute a microniche abundance matrix (MAM) whose *n,p*-th entry is the fraction of patches from sample *n* in microniche *p* (Fig. 1D). It then uses the MAM to perform both a “global” and a “local” association test. The global test tests for the simple presence of any association between the spatial data and case-control status; the local test tests each microniche to determine which microniches are driving the association (Fig. 1E).

To perform the global test, VIMA leverages the widespread correlation in abundance among microniches. This correlation results both from microniches being anchored on physically proximal patches and, more importantly, from microniches capturing similar biology across separate samples. VIMA applies PCA to the MAM to discover microniches whose abundances change in a coordinated fashion across samples. It then tests the MAM principal components (MAM-PCs) for association to case-control status across samples using a linear model. To ensure robust testing, VIMA chooses the number of MAM-PCs to include in a data-dependent manner and computes a permutation-based P-value that accounts for this choice (Methods).

To perform the local test, VIMA computes the correlation between the abundance of each microniche and case-control status. We refer to this correlation as the “microniche coefficient” of that patch. Since microniches can be correlated across samples, VIMA estimates empirical false discovery rates (FDRs) for each microniche coefficient by permuting sample labels and re-computing the correlations to obtain null distributions. In both the global and local tests, VIMA can control for sample-level confounders, such as demographic variables and technical parameters (Methods).

VIMA requires minimal parameter tuning and has favorable runtime properties: for a post-QC dataset of 75 samples with 516 million transcripts resulting in 172,000 tissue patches, the full VIMA workflow takes approximately 2 hours on a single GPU, of which only 78 minutes (65%) is GPU-based model training while the rest is non-GPU-based preprocessing. We have released open-source software implementing the method.

### Performance assessment with simulations

To assess VIMA’s performance, we used a published spatial transcriptomics dataset of S=75 post-mortem brain samples from the middle temporal gyrus of N=27 donors with and without dementia profiled with a 140-gene MERFISH panel^32^. We downsampled the dataset to one unique sample per donor. In null simulations designed to assess VIMA’s calibration (type I error), we found that VIMA was indeed well calibrated, with a global P < 0.05 in 9/300 trials (type I error rate of 0.03 +/− 0.0034 at level 0.05; Methods and Supplementary Fig. 1).

**Figure 2:**
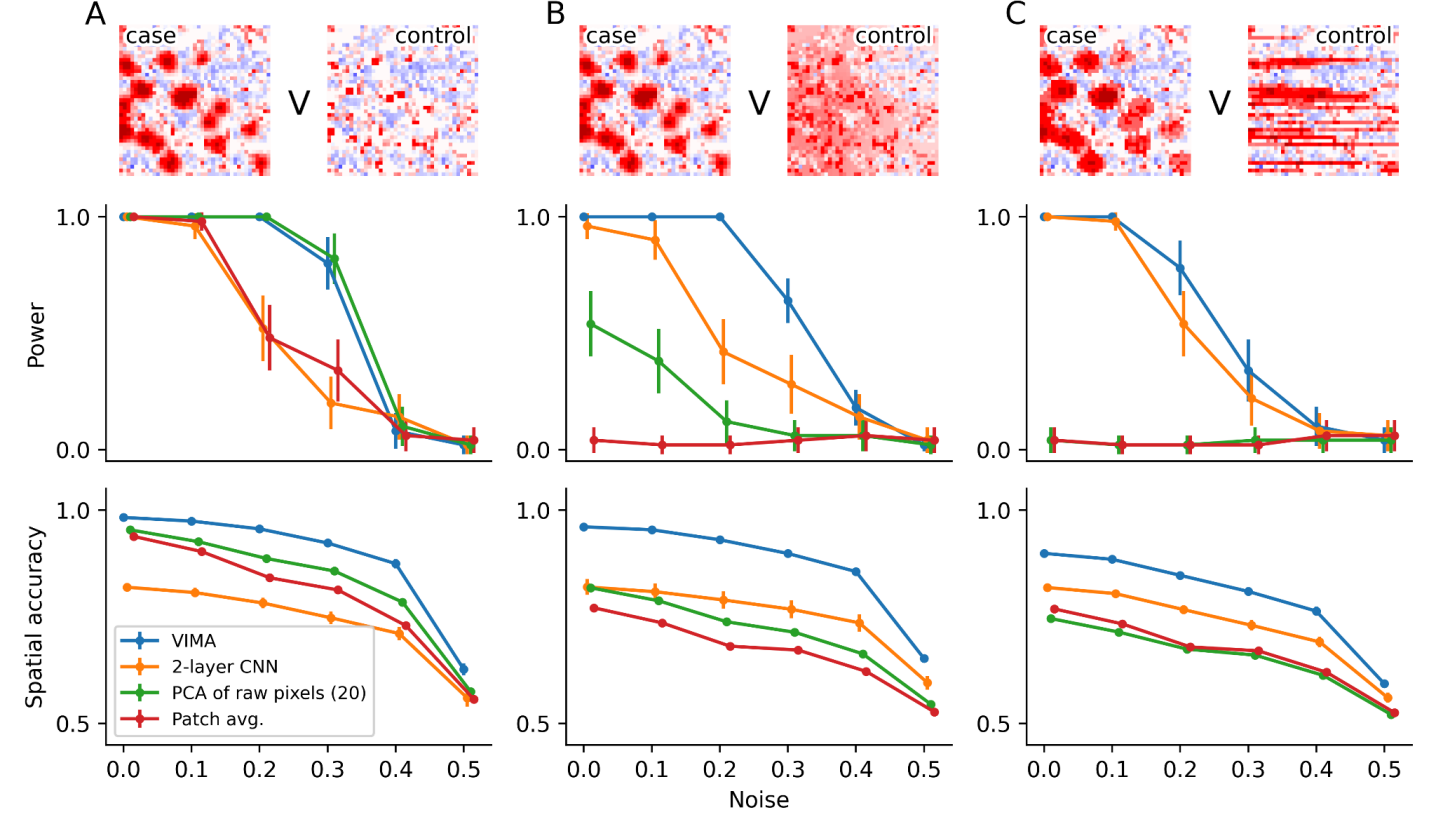
Comparison of VIMA to other methods using a real brain spatial transcriptomics dataset augmented with simulated case-control signals. Each column shows a different signal type spanning **A)** cellular aggregates added to specific cortical layers in case samples vs no modification to control samples, **B)** cellular aggregates added to specific cortical layers in case samples vs diffuse infiltrate added to the same cortical layers in control samples, **C)** cellular aggregates added to specific cortical layers in case samples vs striated tissue structures added to the same cortical layers in control samples. The top row depicts an example tissue patch that has been modified to contain each of the signal types, with the color of each pixel corresponding to intensity of meta-marker 2. The middle row shows the power of each method at level *α* = 0.05 as a function of increasing amounts of noise. The bottom row shows the spatial accuracy of each method at identifying the regions of each sample that contain the case/control signal, as measured by AUROC (Methods).

To assess VIMA’s statistical power (type II error) and its accuracy at spatially localizing case-control differences, we synthetically introduced three realistic types of case-control difference into cortical layer 2/3 of the samples: A) a simple scenario in which we add focal cellular aggregates of a specific cell type to cases but not controls (Fig. 2A), B) a more complex scenario in which we add focal cellular aggregates of a specific cell type to cases and a diffuse tissue infiltrate to controls (Fig. 2B), C) another complex scenario in which we add focal cellular aggregates of a specific cell type to cases and striated tissue structures to controls (Fig. 2C). We measured power at level *α* = 0.05 and measured spatial accuracy using area under the receiver operating curve (AUROC) for classifying patches as case/control-relevant (i.e., in cortical layer 2/3) or not (Methods).

We found that VIMA was well powered across all three simulation scenarios, maintaining power greater than 75% even after adding 20-30% noise across all three signal types (Fig. 2). Similarly, we found that VIMA maintained an accuracy of 75-85% even at 40% noise across the three signal types. VIMA’s local association test also performed well, identifying thousands of significant microniches across a range of noise levels in all three cases (Supplementary Fig. 2).

We next sought to determine which components of VIMA were essential for its strong performance. We did this by formulating several VIMA-like methods that instead of VIMA’s autoencoder represent tissue patches using i) average expression profile across all pixels in each patch, as in the local averaging paradigm, ii) the raw pixel values of each patch represented as a flattened vector and passed through PCA across all patches to reduce dimensionality, and iii) a variational autoencoder based on a two-layer convolutional neural network rather than the more sophisticated ResNet18-like architecture used by VIMA (Methods). We deferred comparisons to approaches based on cell-type abundance to the real datasets, as the computational cost of cell segmentation in our large-scale simulation was prohibitive.

Overall, VIMA substantially outperformed the simpler approaches we formulated above. It was better powered than all of the approaches at detecting the two more sophisticated signal types, and it matched the power of the best-performing alternative approach on the simplest signal type (Fig. 2). Of the comparator methods that we assessed, the patch-wide average expression profiles method was the least well-powered, identifying only the first and simplest of the three signal types with any appreciable power. PCA of the raw pixel values of each patch performed better but was still unable to identify signal (C), while the two-layer convolutional neural network was able to identify all three signal types but with substantially less power than VIMA. VIMA similarly outperformed the comparator methods in terms of accuracy (Fig. 2). We also tried using VIMA’s patch fingerprints in a naive clustering-based case-control analysis framework rather than one based on microniches; this too led to substantial power and accuracy loss (Supplementary Fig. 3). Taken together, these results show that both representing patches using advanced autoencoders and then conducting case-control analysis within the microniche framework improves power and accuracy in identifying spatial signals.

In secondary analyses, we additionally showed that VIMA detects more statistically significant microniches at a specified FDR threshold than the methods based on simpler patch representations (Supplementary Fig. 2), and that the variational penalty in the variational autoencoder underlying VIMA boosts performance but that VIMA’s performance is robust to variation in this parameter spanning several orders of magnitude (Supplementary Fig. 4).

### Capturing tissue features shared across samples using microniches

To examine the properties of VIMA’s patch fingerprints and their associated microniches in a real dataset that is easy to visualize, we applied VIMA to a 7-marker immunohistochemistry (IHC) dataset of S=27 synovial biopsies from N=22 rheumatoid arthritis patients collected by the Accelerating Medicines Partnership Rheumatoid Arthritis/Systemic Lupus Erythematosus (AMP RA/SLE) consortium^31^. (See Supplementary Table 1 for the list of markers profiled.)

**Figure 3:**
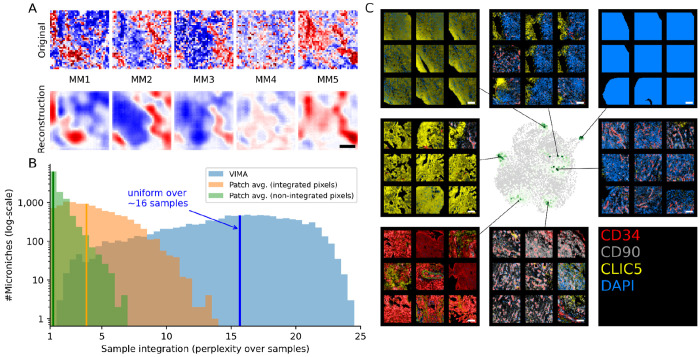
Basic properties of VIMA’s microniches in the RA dataset. **A)** Direct comparison of an example tissue patch (top row) against the reconstruction provided by VIMA’s autoencoder (bottom row). For each of the five meta-markers (MMs), we depict the marker value across the pixels in the original and the reconstructed patch. The meta-marker used to color pixels in each pair of pictures is indicated by the labels MM1, …, MM5. **B)** Histogram of the degree of integration of all the microniches in the dataset as measured by perplexity (exponentiated entropy) of the distribution of each microniche across samples, for microniches generated using either VIMA’s patch fingerprints (blue), naive averaging of the original markers within each patch (orange), and naive averaging of the 5 meta-markers within each patch, which are integrated across samples using Harmony (green). Median perplexity is indicated with a vertical line in each case. To aid interpretation, the arrow indicates that the median perplexity of the VIMA microniches equals the perplexity of a uniform distribution over approximately 16 samples. **C)** A UMAP of all the patches in the dataset generated using the patch fingerprints, together with randomly selected patches drawn from 7 example microniches in the dataset; these microniches are characterized by (clockwise from bottom-left): high CD34 intensity with interspersed CLIC5 signal, high CLIC5 intensity with minimal CD34 signal, high CLIC5 intensity but with a blurrier texture and interspersed DAPI signal, a vertical boundary between CLIC5-high/DAPI-low pixels and CLIC5-low/DAPI-high pixels, a DAPI staining artifact, DAPI-high infiltrate surrounding CD34-high regions that are likely blood vessels, and perivascular CD90-high pixels. All scale bars represent 100um.

The VIMA autoencoder reconstructed the tissue patches with a mean-squared error (MSE) of 0.59. This is a substantial improvement over the MSE of 1 that results from guessing the dataset-wide average for all pixels and the MSE of 0.75 that results from guessing the average expression profile of each patch. Fig. 3A shows an example of a tissue patch from the dataset together with its reconstruction based on the patch fingerprint alone. VIMA’s microniches generally included patches from many different samples: the median sample perplexity among the microniches was 15.7 (for reference, a uniform distribution over *n* samples has a perplexity of *n*; Fig. 3B). This was substantially higher than what we achieved using the local averaging paradigm: microniches based on patchwide average marker intensity had a median perplexity of 1.2, and microniches based on patchwide average meta-marker intensity, which reflects the use of Harmony to remove sample-specific artifacts at the pixel level, had a median perplexity of 3.8 (Fig. 3B, Methods).

On visual inspection, the microniches generated appear to exhibit consistent biological features (Fig. 3C). For example, we observed microniches containing: broad CD34-high staining with small interspersed CLIC5-high areas, likely representing CD34+/CD90− sublining fibroblasts surrounding small collections of lining fibroblasts; large areas of CLIC5-high staining with minimal CD34 signal, indicative of regions of lining fibroblasts; CLIC5-high areas but with a blurrier staining texture and interspersed DAPI signal; a vertical boundary between CLIC5-high regions of lining fibroblasts and CLIC5-low/DAPI-high regions; DAPI saturation that likely represents staining artifact; DAPI-high infiltrate surrounding CD34-high regions that are likely blood vessels; and perivascular CD90-high staining that represents either pericytes or CD90+ lining fibroblasts surrounding blood vessels. These microniches reinforce the potential of VIMA’s approach to aggregate together areas with concordant tissue features across multiple samples in a dataset.

### Identifying dementia-associated tissue structures using spatial transcriptomics data

Alzheimer’s disease is the most common cause of dementia in older patients^38^. Efforts to characterize the molecular signatures of Alzheimer’s dementia using single-cell RNA-seq have identified key cell populations whose aggregate abundance is either increased or decreased in Alzheimer’s patients^32^. However, the question of which multi-cellular spatial structures are characteristic of Alzheimer’s dementia, and of dementia more broadly, has been difficult to study with non-spatial single-cell technologies. To address this, we applied VIMA to a 140-gene MERFISH dataset consisting of S=75 post-mortem medial temporal gyrus samples from N=27 patients, 15 of whom had either Alzheimer’s dementia or an Alzheimers-related dementia^32^, with a total of 516 million transcripts profiled. (See Supplementary Table 2 for the list of genes profiled.) The published analysis of this dataset^32^ used non-spatial single-cell data in a larger sample (N=84) to reveal a negative association between abundance of neurons from cortical layer L2/3 and dementia status. This association has been observed in prior histology-based^39^ and omics-based studies^32,40^ and is also consistent with neuroimaging data suggesting a correlation between the thickness of this layer and measures of cognitive ability^41^.

**Figure 4:**
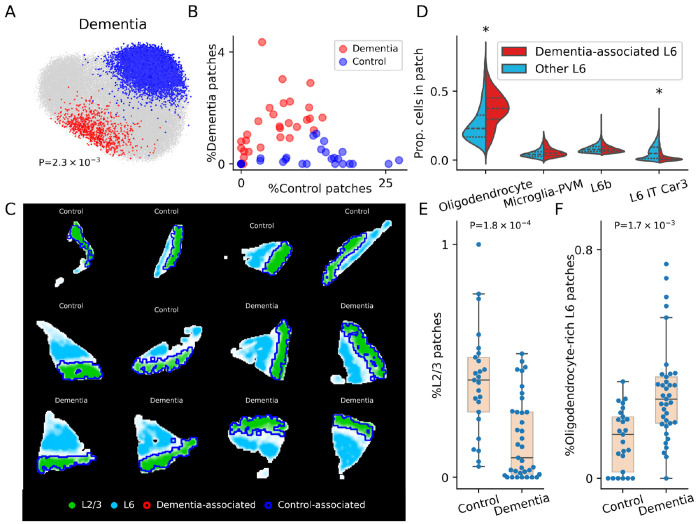
VIMA analysis of Alzheimer’s dataset. **A)** Results of VIMA case-control analysis for dementia versus control: a UMAP of all the patches in the dataset is generated using the patch fingerprints, and the anchor patches for microniches passing the 10% FDR threshold are colored in proportion to the correlation between the abundance of each microniche and dementia status, with red denoting positive correlations and blue denoting negative correlations.The global P-value for the association test is overlaid. **B)** Comparison of fraction of dementia-associated vs control-associated patches in each sample. For each sample, the percent of patches in that sample that anchor microniches significantly positively associated with dementia is plotted against the percent of patches in that sample that anchor microniches significantly negatively associated with dementia. Each sample is then colored by its dementia status. **C)** Representation of a selected subset of samples with green depicting density of layer 2/3 neurons, blue depicting density of layer 6 neurons, contiguous areas of dementia-associated patches outlined in red, and contiguous areas of control-associated patches outlined in blue. **D)** Distributions of cell type proportions of selected cell types among dementia-associated patches and non-dementia-associated layer 6 patches. * denotes statistical significance via sample-level permutation test after corrected for number of cell types tested (Methods and Supplementary Table 3). **E)** Distribution of fraction of layer 2/3 patches in dementia vs control samples, with one-sided Kolmogorov-Smirnov P-value overlaid. **F)** Distribution of fraction of oligodendrocyte-rich layer 6 patches in dementia vs control samples, with one-sided Kolmogorov-Smirnov P-value overlaid.

In our analysis, VIMA detected a significant association to dementia status with many significant microniches, despite the lower sample size of the spatial data (global P=2.3e-3; 8,039 out of 70,898 microniches significant at FDR 10%; Fig. 4A). Moreover, the association appears quite strong: the percent of dementia-associated vs control-associated patches in each sample separated dementia samples from control samples very well (Fig. 4B). In contrast, we were unable to pick up this association at all with the local averaging paradigm: we obtained null results when we represented patches by cell-type proportions generated using cell segmentation and typing (Methods) and applied either a cluster-based case-control framework (0 significantly associated clusters; Supplementary Fig. 5) or microniche-based case-control framework (global P=0.14 with no significant microniches). Similarly, we obtained null results when we used the same procedures but instead represented patches using average expression profiles generated by averaging the meta-markers within each patch (0 significantly associated clusters and global P=0.73 with no significant microniches, respectively; Methods and Supplementary Fig. 6). We also were unable to recover any patches positively associated with dementia when we directly fed VIMA’s patch fingerprints into a cluster-based case-control framework, though in this case we did find a cluster of patches negatively associated with dementia (Methods and Supplementary Figs. 7 and 8). To quantify the power gain of a spatial analysis with VIMA as opposed to a case-control analysis based only on non-spatial single-cell data at the same sample size, we also conducted a case-control analysis just using the sample-wide cell-type proportions for each sample. This analysis was also null (Supplementary Fig. 9), suggesting that the dementia-versus-control difference found in the original study using scRNA-seq alone truly required the higher sample size used in the original study.

The associated patches have clear relationships to cortical architecture and contain both established and novel biological insights. The control-associated patches co-localize very well with cortical layers 2 and 3 (Fig. 4C), suggesting thinning of these layers in dementia in agreement with the literature described above. The dementia-associated patches lie within cortical layer 6, but they only occupy a subset of it (Fig. 4C). To answer the question of what distinguishes the dementia-associated patches from the other layer 6 patches, we looked for cell type abundance differences between the two sets of patches. We found that the dementia-associated patches contain substantially more oligodendrocytes (1.63x enrichment, P<1e-4 by donor-level permutation test) and fewer L6 IT Car3 neurons (0.15x depletion, P<1e-4 by donor-level permutation test) than the other layer 6 patches (Fig. 4D and Supplementary Table 3). We verified that the dementia-associated patches do not contain different proportions of L6b neurons (Fig. 4D and Supplementary Table 3), making it unlikely that the other cell type differences are driven by VIMA identifying, e.g., patches that are more vs less typical of layer 6. To further ensure that our interpretation of both the dementia and control associations was correct, we reproduced them by manually annotating each patch with a binary label denoting whether it has a high abundance of layer 2/3 neurons as well as a binary label denoting whether it has a high abundance of layer 6 neurons and a high abundance of oligodendrocytes (Methods). When we counted the fraction of patches in each sample meeting these criteria and stratified by dementia status, we reproduced both the depletion of layer 2/3 in dementia (P=1.8e-4 by one-sided Kolmogorov-Smirnov test, Fig. 4E) and the enrichment in oligodendrocyte-rich layer 6 neurons in dementia (P=5.0e-3 by one-sided Kolmogorov-Smirnov test, Fig. 4F). Oligodendrocytes have recently been suggested to play a role in the generation of amyloid in the cortex^42^; our results reinforce the potential importance of this cell type for dementia and perhaps even raise the possibility that the newly discovered amyloidogenic activity could be localized to a specific cortical layer.

### Identifying effects of TNF inhibition on lymphoid aggregates in ulcerative colitis using CODEX data

Ulcerative colitis is a chronic inflammatory disease characterized by inflammation and ulceration of the colonic mucosa that leads to symptoms such as diarrhea, abdominal pain, and rectal bleeding^43^. In the last decade, inhibition of tumor necrosis factor (TNF) has emerged as a cornerstone of treatment^43^. However, not all patients respond to TNF inhibition, and the molecular determinants of this heterogeneity are not known^44^. To identify the spatial consequences of TNF inhibition in the colon, we applied VIMA to a 52-marker CODEX^8^ dataset of S=42 colonic biopsies from N=34 patients, 29 of whom had ulcerative colitis^18^ and the rest of whom were healthy. (See Supplementary Table 4 for the list of markers profiled.) The published analysis of this dataset^18^ used a detailed, cell-type based local averaging workflow requiring segmentation of each sample into cells, clustering of the cells into cell types, and clustering of spatial regions on the basis of cell-type abundance into “cellular neighborhoods”. In one specific subgroup of UC patients – those with intermediate-grade colonic inflammation – this analysis strategy revealed a subtle negative association (P<0.01) between treatment with TNF inhibition and the presence of one of the 10 cellular neighborhoods tested that was annotated as containing lymphoid aggregates.

**Figure 5:**
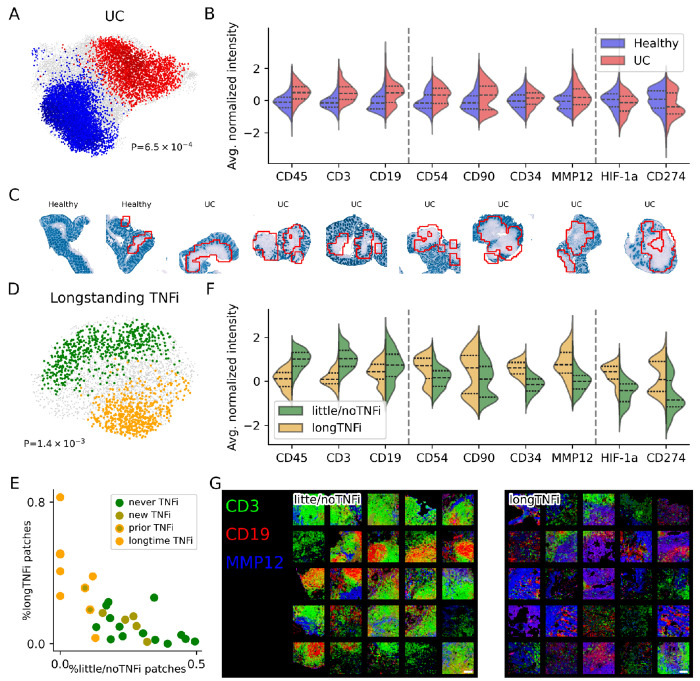
VIMA analysis of UC dataset. **A)** Results of VIMA case-control analysis for UC versus healthy: a UMAP of all the patches in the dataset is generated using the patch fingerprints, and the anchor patches for microniches passing the 10% FDR threshold are colored in proportion to the correlation between the abundance of each microniche and UC status, with red denoting to positive correlations and blue denoting negative correlations. The global P-value for the association test is overlaid. **B)** Distributions of average intensity per patch of selected markers among UC-associated and healthy-associated patches. The selected markers are grouped using dotted lines according to the relationship of their directions of effect to those in panel (F). **C)** Selected samples shown with cytokeratin staining in blue and contiguous areas of UC-associated patches outlined in red. **D)** Results of VIMA analysis of UC-associated patches only, comparing the samples with longstanding TNF inhibition (longTNFi) to those with no TNF inhibition, prior TNF inhibition only, or recent TNF inhibition only (little/noTNFi), with gold denoting positive correlation to longTNFi status and green denoting negative correlation. The global P-value for the association test is overlaid. **E)** Comparison of fraction of longTNFi-associated vs little/noTNFi-associated patches in each sample. For each sample, the percent of patches in that sample that anchor microniches significantly associated with longTNFi is plotted against the percent of patches in that sample significantly associated with little/noTNFi. Each sample is then colored by its detailed TNFi status. **F)** Distributions of average intensity per patch of selected markers among TNFi-associated and non-TNFi-associated patches. The selected markers are grouped using dotted lines as follows: markers that are higher in UC compared to control and in little/noTNFi compared to longTNFi (left), markers that are higher in UC compared to control but lower in little/noTNFi compared to longTNFi (middle), and markers that are lower in UC compared to control and lower in little/noTNFi compared to longTNFi (right). **G)** Randomly selected patches from the little/noTNFi-associated patches (left) and the longTNFi-associated patches (right).

In our re-analysis, we asked whether VIMA could detect spatial signatures of both UC and TNF inhibition in a more statistically robust fashion and without the manual steps of cell segmentation, cell clustering, cell neighborhood clustering, selection of specific patient subgroups, and subsequent testing of multiple targeted hypotheses in those subgroups. We first used VIMA for the comparatively easier task of distinguishing UC samples from healthy samples and found a strong association with many significant microniches (global P=6.5e-4; 8,918 of 21,359 microniches significant at FDR 10%; Fig. 5A). As expected with an inflammatory disease, VIMA found that the patches anchoring the UC-associated microniches had significantly higher average levels of immune cell markers such as CD3 (T cells), CD19 (B cells), and CD45 (most immune cells; Fig. 5B). We also found that the UC-associated tended to lie deep to the epithelial cells, consistent with known immune-cell infiltration of these layers in ulcerative colitis^45,46^ (Fig. 5C).

To define spatial features of TNF inhibition, we then restricted our attention to the UC samples only, and to the patches in those samples that anchored microniches that were significantly positively associated with UC status. We then used VIMA to conduct a case-control analysis among these patches only comparing UC patients exposed to longstanding TNF inhibition (“longTNFi”) to UC patients with either prior but not current TNF inhibition, current but not prior TNF inhibition, or no TNF inhibition at all (“little/noTNFi”; Methods). Strikingly, we found a strong association (global P=1.4e-3; 1,189 of 2,994 microniches significant at FDR 10%; Fig. 5D). The proportion of patches in each sample that were significantly longTNFi-associated versus little/noTNFi-associated strongly stratified samples by TNF status: we observed a gradient with patients exposed to longstanding TNF inhibition on one end, followed by patients with prior but not current TNF inhibition, then patients with recent initiation of TNF inhibition, and finally patients who have never been on TNF inhibition (Fig. 5E).

The longTNFi-associated microniches had some features in common with healthy tissue and other features in common with UC tissue. For example, they had lower levels of traditional B- and T-cell markers similar to the healthy-associated microniches, but they also had higher levels of fibroblast, macrophage, and mesenchymal markers such as CD54, CD90, CD34, and MMP12 (Fig. 5F), similar to the UC-associated microniches (Fig. 5B). We verified these differences at the level of microniche correlations as well (Supplementary Fig. 10). We next examined the spatial relationships among the markers *within* the associated tissue patches. We found that many of the little/noTNFi-associated patches were characterized by B-cell aggregates with surrounding T-cells, consistent with lymphoid aggregates, while the longTNFi-associated patches showed a spatially disorganized pattern of mesenchymal markers intermixed with scant B- and T-cells (Fig. 5G). Overall, these results suggest that TNF inhibition strongly affects colonic tissue architecture, perhaps persistently even after treatment, and that TNF inhibition specifically reduces lymphoid aggregates while upregulating mesenchymal and fibroblast activity.

We were unable to recover the above signals within the local averaging paradigm on this dataset. We obtained too zero or nearly zero UC-associated patches when we represented each patch using average expression profiles and applied either a cluster-based case-control framework (0 significantly UC-associated clusters; Supplementary Fig. 11) or a microniche-based case-control framework (only 3 significant UC-associated microniches at FDR 10%; Methods). The average expression-based representation was therefore not expressive enough to enable an analysis of the effects of TNF inhibition. For comprehensiveness, we also analyzed the data using VIMA’s patch fingerprints in a cluster-based rather than microniche-based association testing framework and again found no significantly UC-associated clusters (Supplementary Figs. 12 and 13). Thus, VIMA’s ability to flexibly model the spatial relationships of the markers within each patch and then to aggregate patches into microniches allows it to powerfully extract biologically meaningful signal from this dataset.

### Identifying spatial features of rheumatoid arthritis subtypes using immunohistochemistry data

Rheumatoid arthritis is an inflammatory arthritis affecting up to 1% of the population in which immune infiltration alters the synovium of the joints, leading to tissue destruction^47^. Our group recently demonstrated that total cell-type proportions in synovial tissue as measured by single-cell RNAseq can be used to subphenotype rheumatoid arthritis patients into six different “cell-type abundance profiles” (CTAPs). Importantly, these subphenotypes have some predictive power for treatment response^31^; however, their spatial correlates are not yet known. To identify these spatial correlates, we returned to the 7-marker immunohistochemistry (IHC) dataset of S=27 synovial biopsies from N=22 rheumatoid arthritis patients collected by the Accelerating Medicines Partnership Rheumatoid Arthritis/Systemic Lupus Erythematosus (AMP RA/SLE) consortium^31^. (See Supplementary Table 1 for the list of markers profiled.) The samples in this dataset were each labelled with a CTAP. The CTAPs labels are T, TB, TM, TF, EFM, and F and reflect a preponderance of various combinations of T cells (T), B cells (B), fibroblasts (F), and myeloid cells (M).

**Figure 6:**
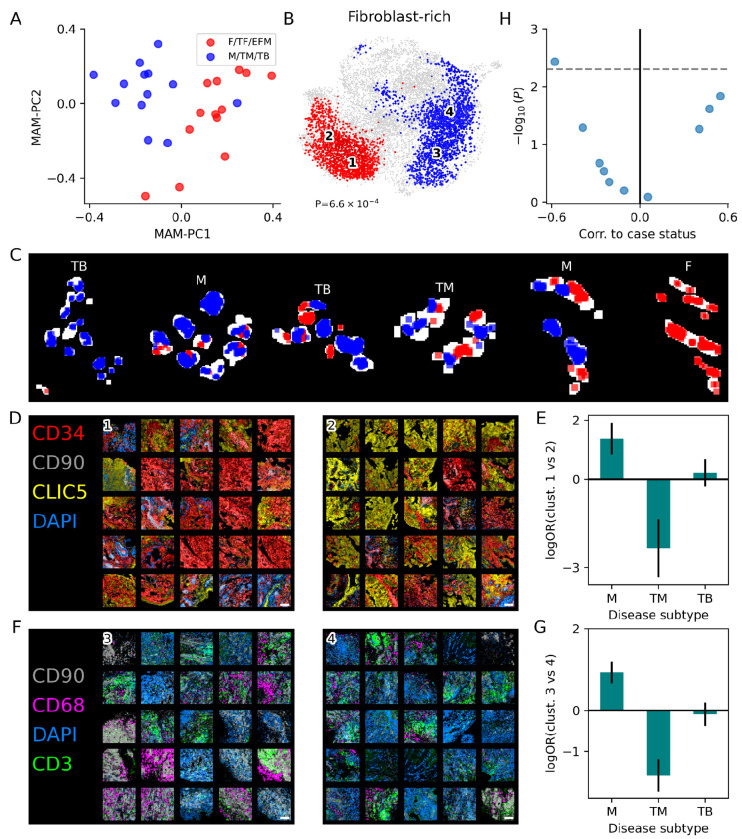
VIMA analysis of disease subtypes in the RA dataset. **A)** plot of MAM-PC1 vs MAM-PC2, with each dot representing a sample and samples colored by RA subtype (CTAP) as determined using a larger scRNA-seq dataset. F: fibroblast, TF: T-cell/fibroblast, EFM: endothelial/fibroblast/myeloid, M: myeloid, TM: T-cell/myeloid, and TB: T-cell/B-cell. **B)** Results of VIMA case-control analysis for fibroblast-rich (F/TF/EFM) versus fibroblast-poor (M/TM/TB) subtypes: a UMAP of all the patches in the dataset is generated using the patch fingerprints, and the anchor patches for microniches passing the 10% FDR threshold are colored in proportion to the correlation between the abundance of each microniche and the fibroblast-rich status, with red denoting positive correlations and blue denoting negative correlations. The centroids of the two subclusters of each of the positive and negatively associated patches, respectively, are indicated with numbers. The global P-value for the association test is overlaid. **C)** A subset of 6 samples from the dataset exemplifying both spatial homogeneity in local fibroblast rich/poor status with a sample (the leftmost and rightmost samples) and spatial heterogeneity in fibroblast rich/poor status within a sample (middle samples), with disease subtype indicated above each sample. Each patch in each sample is colored red if it is fibroblast-rich-associated by VIMA or blue if it is fibroblast-poor-associated. **D)** Randomly selected patches from each of the two clusters of fibroblast-poor-associated patches. **E)** Log-odds ratios among the fibroblast-poor-associated patches for belonging to cluster 1 (positive log-odds) vs cluster 2 (negative log-odds) as a function of disease subtype, with 95% confidence intervals; results for other subtypes are shown in Supplementary Fig. 16. **F)** Randomly selected patches from each of the two clusters of fibroblast-rich-associated patches. **G)** Log-odds ratios among the fibroblast-rich-associated patches for belonging to cluster 3 (positive log-odds) vs cluster 4 (negative log-odds) as a function of disease subtype; there were too few fibroblast-rich associated patches in the samples with disease subtypes F, TF, and EFM to conduct this analysis for those subtypes. **H)** A volcano plot showing the results of a case-control analysis conducted by clustering the patches using their patch fingerprints and then using a T-test to test for association of each cluster. The Bonferroni significance threshold is shown with a dotted line. Scale bars represent 100um throughout.

We used VIMA to look for spatial features of these disease subtypes focusing on the key emerging role of fibroblasts^48,49^, which have recently been recognized as functionally heterogeneous within the RA synovium and which correlate with poor response to treatment^31^. We first examined the MAM-PCs, finding that the first two MAM-PCs nearly perfectly separate the samples assigned to fibroblast-rich CTAPs (F, TF, EFM) from those assigned to the other CTAPs (TB, TM, M; Fig. 6A). On formal association testing, VIMA identified a strong case-control difference between the fibroblast-rich and fibroblast-poor samples (P=6.6e-4; 3,669 of 10,345 microniches significant at FDR 10%; Fig. 6B). Reassuringly, the fibroblast-rich-associated microniches were higher in CLIC5 (a marker of lining fibroblasts) and the fibroblast-poor-associated microniches were higher in CD3 (T cells), CD68 (macrophages), and DAPI (nucleated cells; Supplementary Fig. 14). Importantly, however, we found that the distribution of these cell types was not spatially uniform within samples. For example, even though each sample was classified by the non-spatial single-cell data as either overall fibroblast-rich or overall fibroblast-poor, the spatial distribution of significantly associated patches from VIMA’s analysis showed that a single sample can in fact contain both fibroblast-rich-like regions and fibroblast-poor-like regions (Fig. 6C). We conclude from this that some cell types form spatially organized communities in specific regions of tissue that have distinct types of biology and are not adequately captured by sample-wide cell-type abundances. This spatial characterization advances our understanding of how key cell types in the synovium form spatial communities to create disease states and of how much biological heterogeneity can exist within even a single patient.

VIMA also found clear biological differences within the tissue patches associated with fibroblast-rich and fibroblast-poor status, respectively. For example, the patches associated with fibroblast-poor status clustered into two subtypes (Fig. 6B). The first is highly cellular, characterized by strong DAPI staining intermixed with a T-cell infiltrate (Fig. 6D, cluster 1; Supplementary Fig. 15). The second is characterized by spatial proximity of areas high in the macrophage marker CD68 and areas high in the sublining fibroblast marker CD90 (Fig. 6D, cluster 2; Supplementary Fig. 15). These two clusters were differentially abundant between the M and TM CTAPs, with cluster 1 being more prevalent in the M CTAP (log-odds-ratio −0.9, 95% confidence interval [−1.2,0.66]) and less prevalent in the TM CTAP (log-odds-ratio 1.6, 95% confidence interval [1.2,2.0]; Fig. 6E). (The TB CTAP did not show a significant enrichment, and none of the fibroblast-rich CTAPs had enough patches in these clusters to perform a statistical test.) Similarly, the fibroblast-rich-associated patches also clustered into two subtypes (Fig. 6B). The first is characterized by strong staining of CD34, a marker of sublining fibroblasts and endothelial cells (Fig. 6F, cluster 3; Supplementary Fig. 16). The second is characterized by strong staining of CLIC5, a marker of lining fibroblasts (Fig. 6F, cluster 4; Supplementary Fig. 16). Interestingly, these two clusters were differentially abundant among the CTAPs (Fig. 6G), with cluster 3 being more prevalent in samples from the M CTAP (log-odds-ratio 1.4, 95% confidence interval [0.84,1.9]) and less prevalent in samples from the TM CTAP (log-odds-ratio −2.3, 95% confidence interval [−3.28,−1.32]; Fig. 6G). (Cluster 1 was also enriched in the EFM CTAP and depleted in the TF CTAP, though these effects are smaller in magnitude; Supplementary Fig. 17.) Overall, these findings suggest that the M CTAP may be characterized by vascularization and macrophage/sublining-fibroblast interactions whereas the TM CTAP may be characterized by an expanded lining and an as-yet uncharacterized immune infiltrate with a relationship to T cell activation. VIMA’s ability to spatially characterize the tissue patches in this dataset provides a path toward a clearer elucidation of the biology driving these disease subtypes.

The local averaging paradigm was not able to robustly recover even the difference between fibroblast-rich and fibroblast-poor status. For instance, representing patches using patchwide average expression and then clustering them and testing clusters for association yielded no significant associations (Supplementary Fig. 18). Similarly, representing patches using patchwide average expression and then applying VIMA’s microniche framework found a significant global P-value (P=0.0042) but no significant microniches at FDR 10% consistent with the poor integration shown in Fig. 3B. For comprehensiveness, we additionally analyzed the data in a cluster-based rather than microniche-based association testing framework but still using VIMA’s patch fingerprints (Supplementary Fig. 19). This analysis identified only one significantly associated cluster (adjusted P=0.031) containing 1,448 fibroblast-poor-associated patches and did not identify any fibroblast-rich-associated patches (Fig. 6H).

## Discussion

Here we introduced VIMA, a method for flexible and accurate case-control analysis of spatial molecular datasets that can be applied to a broad range of modalities from immunohistochemistry to many-marker protein modalities and spatial transcriptomics. VIMA uses a variational autoencoder to extract a numerical fingerprint for each patch of tissue in a dataset that quantitatively summarizes the biology of that patch. It then uses the fingerprints to define small, non-discrete groups of highly similar patches called microniches, and asks whether the abundance of these microniches correlates with case-control status. The end result is 1) a fingerprint for each patch, 2) a microniche for each patch describing patches across multiple samples that are highly similar to that patch, 3) a global P-value for association to case-control status, and 4) a subset of the microniches that drive that association at a specified false discovery rate. VIMA does this while accounting for sample-specific artifacts, allowing for inclusion of covariates, requiring minimal parameter tuning, and running efficiently even on datasets with 50-100 samples and hundreds of millions of transcripts. VIMA is different from the deep learning-based methods that have become popular in digital pathology^50,51^, which typically are trained on thousands or even millions of patient samples and focus on patient-level prediction of disease status in a clinical setting rather than defining the specific spatial structures that contribute to that disease in a much smaller research cohort.

We showed in large-scale simulations that VIMA has superior power to detect case-control differences and superior spatial accuracy at localizing them across a range of signal types compared to alternative approaches that either do not use deep learning to represent patches or do not use microniches. We then applied VIMA in three datasets each from a different spatial modality. In each case, VIMA detected novel biological signals, including: a dementia-associated tissue niche consisting of oligodendrocyte-rich areas in cortical layer 6, a statistically robust signature of TNF inhibition in ulcerative colitis characterized by replacement of lymphoid aggregates with disorganized mesenchymal activity, and previously unknown spatial characteristics of rheumatoid arthritis disease subtypes.

Unlike the dominant paradigm of local averaging, in which spatial regions are summarized either using average cell-type abundances or average transcriptional profiles across cells, VIMA’s approach treats the spatial data directly as a set of images and does not rely on segmentation into cells, cell types, and tissue regions. This has the advantage of easily accommodating a broad range of spatial modalities, avoiding the need for error-prone discretization that may not accurately model the data, and reducing the number of user-dependent parameter choices (e.g., number of cell types, tolerance of cell segmentation, etc.). It also allows for simple integration of modalities if more than one is available: each modality’s marker/gene channels can simply be concatenated with the others. As our understanding of this approach improves, it may become possible to also utilize pre-trained models, such as the foundation models that exist today for processing hematoxylin and eosin (H&E) data^50,51^, to boost performance further.

VIMA has several limitations. First, due to its signal type-agnostic approach, VIMA may be less powerful than bespoke methods at lower sample sizes when the signal type in question matches the method being used. Second, although VIMA runs relatively quickly, this speed requires a GPU and using VIMA without one can be time-consuming; this can be ameliorated by using multiple CPUs, but obtaining enough CPUs to match one GPU may be possible only on a computing cluster. Third, although VIMA’s performance appears robust to choices of many of its parameters, we do feel that dramatically different choices of patch size (e.g., 40um vs 400um in length) and dimensionality of the latent space of the autoencoder could lead to emphasis of different types of signals; finding data-dependent ways to choose these parameters is an important direction for future work. Fourth, because neural network training is optimized for speed, hardware race conditions can cause two training runs on identical hardware and with identical input data to yield different models even when all random seeds are set identically. We have observed this behavior with VIMA, and defer to future work the task of either ensuring full reproducibility across training runs or of integrating multiple training runs that produce slightly different outputs. Fifth, since VIMA’s output is not a set of defined and manually annotated tissue niches, interpreting the results of a VIMA analysis can be non-trivial; we maintain, however, that such interpretation is always done, whether before or after the case-control analysis is performed, and that waiting to do it until the spatial regions of interest are delineated may be advantageous.

Despite these limitations, VIMA is a sensitive and accurate way to identify disease-relevant spatial features across a wide range of spatial modalities that is unique in leveraging the power of deep learning within a rigorous and flexible statistical framework. As spatial molecular datasets grow in sample size and dimensionality, methods that are able to give a detailed picture of variation at the sample level will become increasingly important for translating these unprecedented datasets into advances in our understanding of disease.

## Methods

### Variational Inference-based Microniche Analysis

#### Intuition

Variational inference-based microniche analysis is built on two concepts: 1) the patch fingerprint, which is a numerical representation of a tissue patch learned by a variational autoencoder that can be used to quantitatively assess the similarity of pairs of patches, and 2) the microniche, which is a small set of tissue patches from multiple samples that are biologically very similar to each other. Broadly, our method proceeds by using the patch fingerprints to construct the microniches and then finding case-control differences by searching for microniches whose abundance correlates with case status in a statistical hypothesis testing framework.

In the remainder of this section, we establish notation and then proceed to provide a detailed description of 1) how VIMA pre-processes the data, with attention to minimizing pixel-level batch effects, 2) how VIMA extracts patch fingerprints using a variational autoencoder while accounting for spatial batch effects, 3) how VIMA defines microniches, and 4) how VIMA conducts statistical association testing on the microniches.

#### Notation and assumptions

Let $\left\{S_{1},\ldots ,S_{N}\right\}$ be a set of *N* spatial molecular samples with *M* different markers or genes measured for each sample. We assume that each sample has been rasterized into pixels of size *r* x *r*, and we represent each sample $S_{n}$ as a tensor of shape $\left(X_{n},Y_{n},M\right)$ whose (x,y,m)-th entry is either the intensity of marker *m* in the *x,y*-th pixel (for non-transcript-based modalities) or the total count of gene *m* in the *x,y*-th pixel (for transcript-based modalities). For ease of exposition, we occasionally refer below to this number as intensity even for transcript-based modalities. In all analyses in this paper, we set *r* to be 10 micrometers; however, smaller values are in principle possible and could reveal aspects of tissue architecture that play out on smaller length scales.

#### Pre-processing and pixel-level batch correction

VIMA pre-processes data with the goals of a) differentiating tissue from background, b) reducing a potentially large number of markers into a more manageable set of “meta-markers”, each of which is a linear combination of the original markers, and c) removing unwanted sample- or batch-specific artifacts from the meta-markers.

To accomplish (a), a dataset-dependent number is computed for each pixel summarizing the total amount of signal in that pixel: this may be the total number of transcripts in that pixel (for transcript-based modalities), the sum of all marker intensities divided by the sum of the intensities of all negative control stains (for non-transcript-based modalities), etc. VIMA then uses this number to segment each slide into foreground (i.e., tissue) and background. For transcript-based modalities, VIMA simply applies a threshold of 10 transcripts per pixel. For non-transcript-based modalities, in which non-specific background staining is a prominent feature, VIMA instead uses Otsu thresholding^52^. Once the foreground is defined, all background pixel intensities are set to 0 for all markers.

To accomplish (b), VIMA first log-normalizes all non-empty pixels: it computes the median total intensity *q* of all the pixels in the dataset and normalizes each pixel *p* according to the formula

$${\tilde{p}}_{m}=\log\left(q\frac{p_{m}}{p_{1}+\cdots +p_{M}}\right).$$

For each pixel, it then averages the intensity values of any non-empty pixels in the 5x5 square centered at the pixel in question, and it performs PCA on the resulting “meta-pixels”. This PCA yields a set of PCs defined across all the markers. VIMA uses these loadings to then convert each of the original normalized pixels from a length-*M* vector of raw markers to a length-*K* vector of meta-markers defined by the loadings of the top *K* PCs. The default value of *K* is 10, but when a dataset contains fewer than 10 markers, we use *K*=5 instead.

To accomplish (c), VIMA removes sample-specific and batch-specific artifacts by applying the Harmony algorithm^33^ with default parameters to the meta-marker representation of all the non-empty pixels in the dataset.

#### Featurization of patches using variational autoencoder

Once the pixels are pre-processed, VIMA converts each sample into a set of tissue patches of size 40x40 pixels; all patches for which fewer than 20% of the pixels contain tissue (as opposed to background) are discarded.

VIMA then trains a conditional variational autoencoder to learn a *C*-dimensional latent representation from which it can reconstruct the tissue patches, where *C*=100 by default. The loss function that is optimized is:

$$\frac{1}{40\cdot 40\cdot K}\underset{x,y,k}{\sum }{\left(p_{x,y,k}-E_{z_{p}}\left(D{\left(z_{p}\right)}_{x,y,k}\right)\right)}^{2}-\frac{\beta }{2}\underset{c}{\sum }\left(1+\log\left(\sigma^{2}{\left(p\right)}_{c}\right)-\mu {\left(p\right)}_{c}^{2}-\sigma^{2}{\left(p\right)}_{c}\right)$$

where *p* is the original patch (represented as a 40x40x*K* tensor), *D* represents the decoder, μ(p) and $\sigma^{2}\left(p\right)$ are the *C*-dimensional mean and variance respectively of the multivariate normal distribution specified by the encoder as the encoding of *p*, $z_{p}∼𝒩\left(\mu \left(p\right),\sigma^{2}\left(p\right)\right)$ is a sample from that distribution, and β determines the relative weights of the two components of the loss function; a small value of β prioritizes reconstruction while a large value of β prioritizes semantics of the latent space. The loss function is optimized stochastically in the standard way using the reparameterization trick^30^.

The autoencoder’s architecture is shown in Supplementary Fig. 20. This architecture is similar to that of the ResNet^35^ family of neural networks, with the following two modifications. First, it has fewer layers because the patches are of size 40x40 rather than 224x224. Second, the autoencoder is a conditional variational autoencoder^34^, meaning that both the encoder and decoder are given access to sample-level covariates whose influence on the latent representation the user would like to minimize. This is accomplished by allowing the encoder and the decoder to each learn a 4-dimensional embedding $w^{n}$ for sample *n* that is a function of a one-hot encoding of sample ID together with any other covariates supplied. This is converted into four new spatially homogeneous channels by setting all spatial locations in the *i*-th new channel to $w_{i}^{n}$. These new channels are then concatenated to the model’s representation at each layer. In this way, the regularization imposed by the variational penalty incentivizes the encoder to leverage the new channels to remove any sample-specific information, and incentivizes the decoder to then supply that information back during its reconstruction. This method of incentivizing integration by giving the encoder and decoder access to sample-level information has been successfully used in single-cell integration^53^.

In simulation, we show that performance does not strongly depend on β as long as it’s not extremely small. Accordingly, in practice we simply set $\beta =10^{-5}$ with the recommendation that if the patches are not well integrated across samples then β should be increased by one order of magnitude at a time until this is achieved. (We have not had to do this in the datasets analyzed in this paper.)

We train the autoencoder using standard practice, with 80% of the patches used for training and 20% used for validation. We use a batch size of 256 patches, a default learning rate of 10^−3^ with an exponential decay parameter of 0.9 per epoch, KL warmup over the first 5 epochs, and 20 epochs of training with option to extend if the validation loss has not plateaued. These settings were uniform across all datasets processed.

#### Definition of microniches

Once the autoencoder is trained, it is run on all *P* patches in the dataset whose tissue density (the fraction of pixels containing tissue) is greater than some threshold α to produce a *C*-dimensional vector $z_{p}$ for each patch *p*; α is set of 0.5 by default. The embeddings $z_{p}$ are then used to construct a nearest-neighbor graph of all *P* patches using scanpy’s neighbors function^54^.

The microniches are then defined similarly to how transcriptional neighborhoods are defined in our previous work in non-spatial single-cell analysis^36^. That is, we define one microniche for each patch *p* using the similarity implied by the structure of the nearest-neighbor graph: for every other patch *p*’, the degree of belonging to the microniche anchored at patch *p* is the probability that a random walk starting at patch *p*’ will arrive at patch *p* after *s* steps. The transition matrix of the random walk is *A*+*I* where *A* is the adjacency matrix of the nearest neighbor graph and *I* is the identity matrix. The number of steps *s* is chosen adaptively by stopping the random walk once enough microniches are no longer gaining representation from new samples with each step of the walk. This is accomplished by tracking the median kurtosis across microniches of the distribution across samples of each microniche, and stopping when the decrease in this number with each step of the random walk falls below a hardcoded threshold.

Applying the above procedure for each sample in the dataset allows us to build the MAM, which formally is an N×P matrix whose *n,m*-th entry equals the expected fraction of patches from sample *n* that would arrive at patch *p* after *s* steps, that is, the relative abundance of microniche *p* in sample *n*. The MAM can be computed quickly using sparse matrix multiplication since the matrix *A*+*I* is sparse. As with prior work^36^, our software allows for covariates to be projected out of the MAM.

#### Statistical association testing

Given the MAM *Q* and a sample-level attribute of interest *y*, VIMA performs two types of association testing: a *global* association test, which is meant to assess for any aggregate relationship between abundance of the microniches as captured by *Q* and *y*, and a *local* association test, which is meant to find specific microniches whose abundance correlates with *y*.

To conduct the global test, VIMA standardizes the columns of *Q* and performs PCA on it, retaining the first *t* principal components for four different values of *t* ranging from [*N*/50] through min([*N*/50],*N*/5) , where […] denotes the ceiling function. In each case, it then regresses the case-control labels *y* on these *t* principal components and computes an F-test P-value that takes into account the number of principal components used, retaining the minimal F-test P-value $u^{*}$ obtained across the four values of *t*. This P-value is not naively reported because doing so would lead to miscalibration arising from not accounting for the use of four different values of *t*; instead, the P-value is used as a statistic and compared to a null distribution. The null distribution is constructed by creating a large number of random permutations of *y* and performing the same hypothesis testing procedure (including trialing four different values of *t*) with each of these permutations. The statistic $u^{*}$ is then compared to the null distribution to compute a calibrated P-value. To account for the presence of multiple samples from the same donor, which is common in spatial datasets, the permutation scheme used preserves the property that samples from the same donor have the same sample-level attribute.

To conduct the local test, VIMA computes for each patch *p* the correlation $\rho_{p}$ between the abundance of the microniche anchored at *p* with *y*. It then does the same for a large number of random permutations of *y*, constructed as above to account for the potential presence of multiple samples per donor. It then computes, for each potential value of $\rho^{*}$ that might be used as a cutoff for statistical significance, an empirical estimate of the false discovery rate at that threshold. That is, it naively estimates

$$\text{FDR}\left(\rho^{*}\right)=E\left(\frac{\left|\left\{p\;:\;\left|\rho_{p}\right|>\rho^{*}\right\}\right|}{\left|\left\{p\;:\;\left|\rho_{p}^{0}\right|>\rho^{*}\right\}\right|}\right)$$

Where $\rho_{p}^{0}$ is a random variable that represents the correlation between the microniche anchored at patch *p* and a random permutation of *y*.

### Simulations

To assess the calibration, power, and accuracy of VIMA, we performed simulations using a real spatial transcriptomics dataset from the SEA-AD dementia cohort^32^. This dataset consists of S=75 postmortem medial temporal gyrus samples from N=27 patients, 15 of whom had dementia. Each sample was profiled using MERFISH, yielding spatial information about M=140 genes at single transcript resolution, with a total of 516 million transcripts profiled. For computational convenience, we downsampled the dataset to one sample per patient for the simulation study. We processed the dataset following the preprocessing methodology described above resulting in 10 (harmonized) meta-markers per pixel for each sample.

#### Quantifying type 1 error

For the simulation in Supplementary Fig. 1, we generated 300 simulates in which the samples were left unmodified and a random binary case-control label was assigned to each sample. We then quantified type I error at level *α* = 0.05 by computing the fraction of null simulates in which VIMA rejected the null hypothesis.

#### Generation of non-null simulation data

We simulated three non-null scenarios: A) a scenario in which cases have focal cellular aggregates and controls do not, B) a scenario in which cases have focal cellular aggregates while controls have a diffuse infiltrate, C) a scenario in which cases have focal cell aggregates while controls have a striated tissue structure.

To simulate these signals, we first defined a region in each sample corresponding to cortical layer 2/3 by using the annotated cell locations published with the dataset: we identified the pixel location of all layer 2/3 neurons and used standard imaging transforms (closing with radius 40 pixels followed by opening with radius 10 pixels) to define a contiguous region around areas with high density of such pixels. We then identified all pixels lying within the layer 2/3 region annotation whose second meta-marker was above 5 and defined these as *foci* around which we would spike in any new signals. The spiked in signals were: for (a) addition to meta-marker 2 of a Gaussian blur of diameter 7 pixels around each focus in case samples; for (b), addition to meta-marker 2 of a Gaussian blur of diameter 7 pixels around each focus in case samples and a Gaussian blur of diameter 51 pixels around each focus in control samples; for (c), addition to meta-marker 2 of a circle of diameter 7 pixels around each focus in case samples and a rectangle with shape 1x33 pixels around each focus in control samples.

For each non-null signal type, we added increasing amounts of noise by randomly re-assigning the case/control label of a given fraction *h* of the samples for h∈{0,0.1,…,0.5}. We performed 50 simulates for each signal type at each noise level.

#### Methods assessed in non-null simulations

Each of the methods we assessed in simulation was specified by two components: 1) the way in which patches were represented, and 2) the way in which the patch representations were used for case-control analysis.

For (1), we considered the following methods: i) representation of each patch by a 10-dimensional vector obtained by averaging each meta-marker across all pixels in that patch; ii) flattening of each patch, which is a 40x40x10 tensor, into a vector of length 40 · 40 · 10, running PCA on all the patches, and representation of each patch using its value along the top 20 PCs; iii) representing each patch using the latent representation learned by a simple two-layer convolutional variational autoencoder; and iv) representing each patch using the latent representation learned by VIMA’s variational autoencoder. For method (iv), we set *C*, the number of latent dimensions of VIMA’s autoencoder, to 20, and we reduced the number of epochs of training to 10; we made these changes both to speed up computation in order to allow for larger-scale simulations and to match the smaller size of the simulation dataset and the simpler nature of the simulated case-control signals.

For (2), we considered the following two methods: a) case-control analysis using VIMA’s covarying microniche-based approach as defined above, and b) case-control analysis by clustering the patch representations using leiden clustering with a resolution of 1, computing the relative abundance of each cluster in each sample, performing a T-test for each cluster to detect association between the relative abundance of that cluster and the sample labels, and reporting the minimal P-value obtained across all the clusters with Bonferroni correction for the number of clusters tested.

In Fig. 2 and Supplementary Fig. 2, we show results for methods i.a, ii.a, iii.a, iv.a. In Supplementary Fig. 3, we show results for methods iv.a versus iv.b. In Supplementary Fig. 4, we compare iv.a to alternative versions of the same method but with different values of the parameter β defined above, which controls the strength of the variational penalty in VIMA’s variational autoencoder.

#### Figures of merit used to assess non-null simulations

We used three different figures of merit to assess performance in the non-null simulations: 1) power to reject the null hypothesis of no association at level α=0.05, 2) accuracy at identifying the spatial regions of each sample responsible for that sample’s case vs control status, and 3) number of significant microniches detected for methods i.a through iv.a.

For (1), we computed power in the standard way by computing the fraction of simulations in which the null was rejected by each method at each noise level. For (2), we computed accuracy by checking how well the microniche-level scores could be used to identify which patches are truly case-associated, truly control-associated, or truly null. To do this, we computed the average of two different AUROC metrics: the AUROC for using the microniche-level scores to classify layer 2/3 in cases away from the rest of the patches, and the AUROC for using the microniche-level scores to classify layer 2/3 in controls away from the rest fo the patches. If all truly case-associated patches are given positive scores, all null patches are given scores of 0, and all truly control-associated patches are given negative scores, then this metric will equal 1. Conversely, if the microniche-level scores are uninformative then this metric will equal 0.5. For methods i.a through iv.a, we used the microniche-level scores produced by the VIMA software; for method iv.b, we assigned to each patch the T-statistic produced from the T-test applied to the cluster containing that patch. For (3), we used the number of microniches passing the FDR 10% threshold as estimated by VIMA; we did not conduct this analysis for method iv.b as this method does not produce a set of FDR-significant patches.

### Analyses of real data

We analyzed three real datasets: synovial biopsies from patients with RA profiled with immunohistochemistry (S=27 samples from N=22 donors, M=7 markers profiled)^31^; colonic biopsies from patients with and without ulcerative colitis profiled with CODEX (S=42 samples from N=34 donors, M=52 markers profiled)^18^; and post-mortem medial temporal gyrus samples from patients with and without dementia profiled with MERFISH (S=75 samples from N=27 donors, M=140 genes profiled)^32^.

#### Microniche analysis of rheumatoid arthritis dataset

We obtained the RA dataset from the AMP RA/SLE consortium. We applied VIMA with default parameters (except for the use of only 5 meta-markers rather than 10, since there were only 7 markers in total) to generate patch fingerprints and microniches. To compute perplexity for each microniche, we considered each microniche as a distribution over samples and computed the perplexity of that distribution. We then did the same for microniches defined using the naive average marker profile of each patch (referred to as “Patch avg. (non-integrated pixels)”) and using the average meta-marker profile of each patch (referred to as “Patch avg. (integrated pixels)” since the meta-markers for each pixel have been integrated across samples using Harmony).

#### Analysis of Alzheimer’s disease dataset

We downloaded the Alzheimer’s dataset from the SEA-AD website. In addition to spatial data, each patient was also labeled as either having dementia or not. To enable comparison to the local averaging paradigm as instantiated through local cell-type abundances, we performed cell segmentation on the data as described in ref.^55^ and then annotated those cells using the Allen Institute’s MapMyCells tool (https://portal.brain-map.org/atlases-and-data/bkp/mapmycells) using the matching “10x Human MTG SEA-AD (CCN20230505)” reference taxonomy and the “SEA-AD Correlation Mapping” algorithm. As a result, each cell was annotated as having a spatial location and a cell type at several specific cell-type resolutions.

We pre-processed the data with the default preprocessing workflow described above, using the total transcript count per pixel to summarize the total amount of tissue signal in each pixel. This resulted in 10 (harmonized) meta-markers per pixel, and a mask for each sample that defines which pixels are assumed to come from tissue.

We then ran VIMA on the dataset with default settings to check for association to dementia vs non-dementia status, yielding a global P-value for association and a set of significant microniches at FDR 10%.

To determine whether the positively and negatively associated microniches coincide with specific cortical layers, we computed for each patch the proportion of cells in that patch belonging to each cell type. We used this information to create spatial heat maps for the density of the cell types “L2/3 IT” for layer 2/3 and “L6b” for layer 6.

To determine which cell types were most enriched in the dementia-associated patches compared to other layer 6 patches, we computed the proportion of cells in each patch coming from each of the 24 annotated cell types. We then compared the median cell type abundance for each cell type between the dementia-associated patches and the other layer 6 patches. We tested these differences for significance by randomly flipping, for each sample, the association status (dementia-associated vs not) of all of the layer 6 patches in that sample and using this to compute a null distribution for our statistic. This yielded the P-values in Supplementary Table 3.

To confirm our interpretations of the dementia- and non-dementia-associated patches, we used the cell type proportions of each patch to define two types of patches: layer 2/3 patches, defined as those patches with more than 10% of cells belonging to the “L2/3 IT” cell type, and oligodendrocyte-rich layer 6 patches, defined as those patches with more than 5% of cells belonging to the “L6b” cell type and more than 10% of cells belonging to the “Oligodendrocyte” cell type. We then computed the proportion of patches from each of these two categories in each sample and compared the distributions among cases and controls using a one-sided Kolmogorov-Smirnov test.

We also ran the case-control analysis for dementia status using local averaging-type methods and sample-wide averaging-type methods as follows: 1) we represented each patch using a vector containing the proportion of cells in that patch belonging to each of the 24 annotated cell types and either A) clustered the patches using Leiden clustering with the default resolution parameter of 1, tested each cluster for association by computing the correlation between abundance of that cluster and case/control status with a permutation-based null distribution, and applied a Bonferroni correction to account for the number of clusters tested (Supplementary Fig. 5), or B) fed this patch representation into a VIMA-type microniche association testing framework. 2) We represented each patch using its average meta-marker profile and repeated both approach A above (Supplementary Fig. 6) and approach B above using this patch representation. For comprehensiveness, we also fed VIMA’s patch fingerprints into a cluster-based case-control framework similar to A above (Supplementary Figs. 7 and 8). Finally, to compare to a non-spatial analysis of this same dataset, we represented each sample using a vector containing the proportion of cells in that sample belonging to each of the 24 annotated cell types and used correlation to case/control status with permutation-based empirical false discovery rates (Supplementary Fig. 9).

#### Analysis of ulcerative colitis dataset

We obtained the UC dataset in tiff format from the authors of the original study^18^. In addition to spatial data, each patient also has metadata specifying whether they have UC or not, whether they were on a TNF inhibitor at the time of their biopsy, and whether they were on a TNF inhibitor in the past. In addition to the 52 CODEX markers, each sample also had 21 channels consisting of Hoechst stains that were used to register the markers across different rounds of imaging in the CODEX protocol. We noticed that these stains induced substantial inter-sample batch effects so we removed them prior to normalizing the pixel intensities in the dataset.

We pre-processed the data with the default preprocessing workflow described above, using the summed intensity of all the CODEX markers and the nuclear stains to summarize the total amount of tissue signal in each pixel, but using only the CODEX markers for the dimensionality reduction from markers to meta-markers. This resulted in 10 (harmonized) meta-markers per pixel, and a mask for each sample that defines which pixels are assumed to come from tissue.

We then ran VIMA on the dataset with default settings to check for association to UC vs healthy status, removing from the case-control analysis (but not from the training of the autoencoder) any sample that either was on a TNF inhibitor or had been on one in the past in order to maximize the contrast between ulcerative colitis and healthy tissue. This resulted in a global P-value for association as well as a set of microniches significantly associated (both positively and negatively) with UC status at FDR 10%.

To assess which markers have higher average intensity in the UC-associated patches, we computed the average intensity of each marker in each patch. We then estimated the distribution of each marker across all the UC-associated patches identified by VIMA as well as the healthy-associated patches.

To identify patches typical of treatment with TNF inhibition, we took only the patches found by VIMA to have a significant positive association with UC in the UC patients and re-built a new nearest neighbor graph using the patch fingerprints of these patches. We then used this new nearest-neighbor graph to define a new MAM and ran our association testing with this new MAM with a phenotype of “longstanding TNF inhibition” defined as presence of both prior use of TNF inhibition and current use of TNF inhibition in the same patient. We then repeated the same workflow described above to identify markers typical of the positively and negatively associated patches.

To show that the longTNFi-vs-little/noTNFi signature was distinct from the UC-vs-healthy signature, we selected all the patches found to be significantly longTNFi-associated and compared their UC microniche coefficients to those of the patches that were significantly little/noTNFi-associated (Supplementary Fig. 10). If the two signatures were similar, we might expect the longTNFi-associated microniches to have lower correlations to UC on average than the little/noTNFi-associated patches; instead, we saw nearly identical distributions, with the median UC correlation actually being slightly higher among the longTNFi-associated microniches.

We also ran the case-control analysis for UC status using local averaging-type methods as follows: A) We represented each patch using its average meta-marker profile, then clustered the patches using Leiden clustering with the default resolution parameter of 1, tested each cluster for association by computing the correlation between abundance of that cluster and case/control status with a permutation-based null distribution, and applied a Bonferroni correction to account for the number of clusters tested (Supplementary Fig. 11). B) We represented each patch using its average meta-marker profile and analyzed the patches using a VIMA-type microniche approach. For comprehensiveness, we also fed VIMA’s patch fingerprints into a cluster-based case-control framework similar to A above (Supplementary Figs. 12 and 13).

#### Case-control analysis of rheumatoid arthritis dataset

In addition to the immunohistochemistry dataset described above, each patient in the RA dataset had a label – computed using non-spatial single-cell RNA-seq from a separate biopsy in the same patient – indicating the predominant coarse cell types found in that biopsy. This is referred to by the authors of the original study as a cell-type abundance phenotype (CTAP). The CTAP labels are T, TB, TM, TF, EFM, and F and reflect a preponderance of various combinations of T cells (T), B cells (B), fibroblasts (F), and myeloid cells (M). We define F* to be the union of all the fibroblast-containing CTAPs, i.e.,

$${\text{F}}^{*}=\text{F}\cup \text{TF}\cup \text{EFM}.$$

We pre-processed the data with the default preprocessing workflow described above, using the ratio between the summed intensity of all 7 markers and the intensity of the auto-fluorescence channel to summarize the total amount of tissue signal in each pixel. After log-normalization, PCA across meta-pixels, and harmony as described above, this resulted in 5 (harmonized) meta-markers per pixel, and a mask for each sample that defines which pixels are assumed to come from tissue.

We then ran VIMA on the dataset with default settings, except that we set *α* (the fraction of pixels in a patch that must be non-empty for VIMA to include it in the case-control analysis) to 0.8 due to the difficulty accurately distinguishing tissue from nonspecific background staining in this dataset as well as the highly irregular borders of many of the samples. This resulted in a MAM with MAM-PCs, a global P-value for association to F* status, and a set of microniches significantly associated (both positively and negatively) with F* status at FDR 10%.

To assess which markers have higher average intensity in the F*-associated patches, we computed the average intensity of each marker in each patch. We then estimated the distribution of each marker across all the patches identified by VIMA as typical of the F* CTAPs and across all the patches identified by VIMA as typical of the non-F* CTAPs (Supplementary Fig. 14).

To identify subtypes of the F* CTAPs and the non-F* CTAPs, we clustered the positively and negatively associated patches, respectively, into 2 clusters each. We compared average marker levels in the same way that we did for the F*-associated patches (Supplementary Figs. 15 and 16). We then computed the enrichment of each of the F* CTAPs in each cluster as follows. For each of the CTAPs *y* with more than 50 patches represented among the positively associated patches, and for each of the clusters *c*, we calculated the number of positively associated patches in cluster *c* from all samples with CTAP *y*. We then computed the log-odds-ratio, for each CTAP, of a patch from that CTAP belonging to cluster 1 versus cluster 2 of negatively associated patches. We calculated standard errors for the log-odds-ratio using the formula for the asymptotic variance formula for log-odds-ratios computed from contingency tables (1/*a*+1/*b*+1/*c*+1/*d* where *a,b,c,d* are the four counts used). We repeated the same process for the two clusters of positively associated patches (Supplementary Fig. 17).

We also ran the case-control analysis for F* status using local averaging-type methods as follows: A) We represented each patch using its average meta-marker profile, then clustered the patches using Leiden clustering with the default resolution parameter of 1, tested each cluster for association by computing the correlation between abundance of that cluster and case/control status with a permutation-based null distribution, and applied a Bonferroni correction to account for the number of clusters tested (Supplementary Fig. 18). B) We represented each patch using its average meta-marker profile and analyzed the patches using a VIMA-type microniche approach. For comprehensiveness, we also fed VIMA’s patch fingerprints into a cluster-based case-control framework similar to A above (Supplementary Fig. 19 and Fig. 6H).

## Supplementary Material

Supplement 1

## Data Availability

All data analyzed during this study are available via three previously published articles^18,31,32^.
