# Supplementary material for: Powerful and accurate case-control analysis of spatial molecular data with deep learning-defined tissue microniches
Source: bioRxiv. 2025 Feb 8:2025.02.07.637149. Preprint. [Version 1] doi: 10.1101/2025.02.07.637149 (PMC11839118; doi:10.1101/2025.02.07.637149)
Supplement: Supplementary file 1 [file media-1.pdf]

Supplementary Information for "Powerful and accurate case-control analysis of spatial molecular data with deep learning-defined tissue microniches"

Contents

Supplementary Tables **2**

Supplementary Figures **4**

Supplementary Tables

Marker	Comments
DAPI	nuclear stain
CLIC5	lining fibroblasts
CD34	sublining fibroblasts, endothelial cells
HLADR	antigen presentation
CD3	T cells
CD90	sublining fibroblasts, pericytes
CD68	macrophages

Supplementary Table 1: The 7 markers profiled in the RA dataset. For each marker, we include a description of the main associated cell types or states.

ADAMTS3	*COL11A1*	*FRMPD4*	*ITGB8*	*NOS1*	*RBFOX3*	*SOX6*
ADAMTSL1	*CSMD1*	*GAD2*	*KAZN*	*NOSTRIN*	*RFX3*	*STXBP6*
ANK1	*CTSS*	*GALNTL6*	*KCNIP4*	*NPAS3*	*RGS12*	*SULF1*
ASIC2	*CUX2*	*GPC5*	*KCNMB2*	*NPY*	*RGS6*	*SV2C*
ASTN2	*DACH1*	*GRID2*	*KIAA1217*	*NRG1*	*ROBO1*	*TACR1*
ATRNL1	*DCC*	*GRIK3*	*KIRREL3*	*NTNG2*	*ROBO2*	*TAFA1*
BTBD11	*DCLK1*	*GRIN2A*	*L3MBTL4*	*NXPH2*	*RORB*	*TENM2*
CA10	*DCN*	*GRIN3A*	*LAMA4*	*PALMD*	*RYR3*	*TH*
CACHD1	*DGKG*	*GRIP1*	*LAMP5*	*PAX6*	*SATB2*	*THEMIS*
CACNA2D3	*DLC1*	*GRIP2*	*LHX6*	*PDE4B*	*SCUBE1*	*TLL1*
CALB1	*DLX1*	*GRM7*	*LRP1B*	*PDGFD*	*SEMA3E*	*TMEM132D*
CARTPT	*EBF1*	*GRM8*	*LRRC4C*	*PDZD2*	*SEMA6D*	*TMEM255A*
CBLN2	*EGFR*	*HCN1*	*LRRK1*	*PEX5L*	*SLC14A1*	*TNR*
CD22	*ETNPPL*	*HPSE2*	*LUZP2*	*PLCB1*	*SLC24A2*	*TOX*
CD74	*EYA4*	*HS3ST2*	*MEIS2*	*PLD5*	*SLC32A1*	*TSHZ2*
CDH6	*FBXL7*	*HS6ST3*	*MOG*	*PRKG1*	*SLIT3*	*UNC5B*
CHODL	*FEZF2*	*HTR2A*	*NDNF*	*PRRT4*	*SMYD1*	*VIP*
CLSTN2	*FGF12*	*HTR2C*	*NFIA*	*PRRX1*	*SNTB1*	*ZMAT4*
CNTN5	*FGF13*	*ID3*	*NKAIN2*	*PVALB*	*SORCS1*	*ZNF385D*
CNTNAP5	*FOXP2*	*ITGA8*	*NLGN1*	*RBFOX1*	*SORCS3*	*ZNF804A*

Supplementary Table 2: The 140 gene expression markers profiled in the Alzheimer's dataset. Negative control probes are not included in this table.

Cell type	Abund(dementia)	Abund(other)	Enrich.	P	P_{bonf}
L6 IT Car3	0.007407	0.049327	0.15	0.0001	0.0024
Oligodendrocyte	0.374449	0.229730	1.63	0.0001	0.0024
L5/6 NP	0.000000	0.006061	0	0.0005	0.012
L5 ET	0.000000	0.006452	0	0.0012	0.0288
L5 IT	0.004878	0.014925	0.327	0.0104	0.25
L4 IT	0.000000	0.005682	0	0.0236	0.566
L6 IT	0.118919	0.142857	0.832	0.0309	0.742
Sst	0.011765	0.015625	0.753	0.0484	1
L2/3 IT	0.005263	0.006173	0.853	0.14	1
OPC	0.039604	0.033898	1.17	0.148	1
Microglia-PVM	0.051887	0.041237	1.26	0.259	1
L6b	0.074419	0.078652	0.946	0.362	1
Endothelial	0.035897	0.039216	0.915	0.402	1
Pvalb	0.005988	0.007874	0.76	0.405	1
VLMC	0.030864	0.031847	0.969	0.693	1
Astrocyte	0.111650	0.115385	0.968	0.724	1
L6 CT	0.041322	0.040000	1.03	0.822	1
Lamp5 Lhx6	0.006452	0.006410	1.01	0.963	1
Chandelier	0.000000	0.000000	nan	1	1
Lamp5	0.000000	0.000000	nan	1	1
Pax6	0.000000	0.000000	nan	1	1
Sncg	0.000000	0.000000	nan	1	1
Sst Chodl	0.000000	0.000000	nan	1	1
Vip	0.000000	0.000000	nan	1	1

Supplementary Table 3: Cell types enriched in dementia-associated layer 6 patches compared to non-associated layer 6 patches. For each cell type, we list its median abundance (as a fraction of all cells) in dementia-associated layer 6 patches, its median abundance (as a fraction of all cells) in the remainder of the layer 6 patches, the enrichment this corresponds to, the P-value generated by randomly switching the associated and non-associated patches within each sample with 10,000 permutations (Methods), and the Bonferroni-corrected P-value.

Vimentin	CD3	CD137	CD40
CD19	CD126	CD7	FoxP3
CD31	CD66	VCAM-1	CD127
CD85j	CD69	CD49f	CD45RA
TIGIT	CD134	TCRyd	Cytokeratin
Ki67	CD8	CD36	CD104
CD15	CD57	CD16	Podoplanin
HLA-DR	CD56	CD38	CD278
CD152	CD11c	CD1c	CD120b
CD21	HIF-1a	CD123	CollagenIV
CD274	MMP12	CD90	CD117
CD2	CD54	CD45	CD279
CD4	CD34	HLA-ABC	CD5

Supplementary Table 4: The 52 markers profiled in the UC dataset. Hoechst stains and negative control stains are not included in this table.

Supplementary Figures

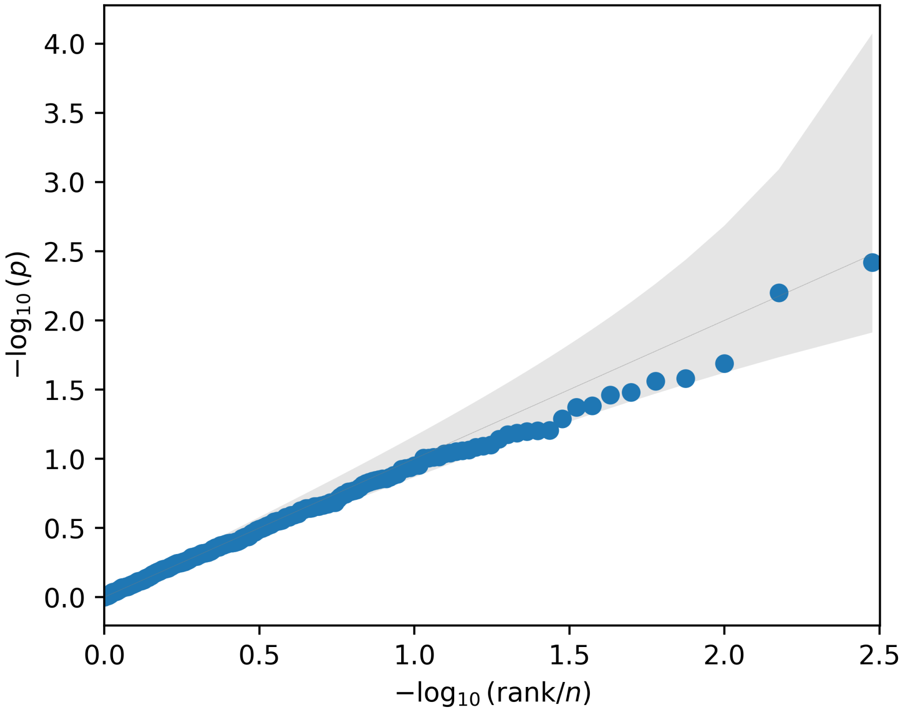

Supplementary Figure 1: Null calibration of VIMA. A Q-Q plot comparing the distribution of VIMA global P-values for case-control association obtained across the 300 null trials with a uniform distribution.

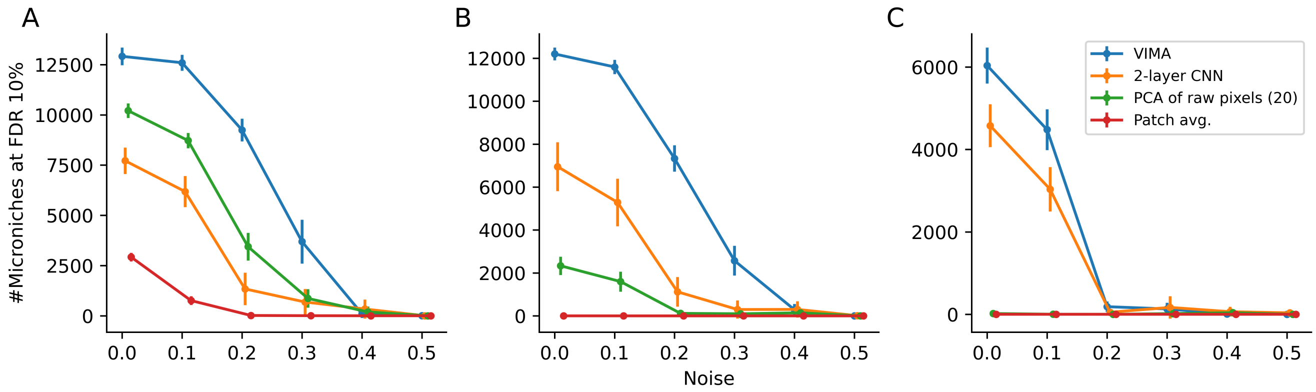

Supplementary Figure 2: Comparison of number of significant microniches at FDR 10% across signal types and patch representations. Each plot quantifies the number of microniches achieving significance at FDR 10% as a function of noise for each of the methods shown in Figure 2, across the three signal types: A) cellular aggregates added to specific cortical layers in case samples vs no modification to control samples, B) cellular aggregates added to specific cortical layers in case samples vs diffuse infiltrate added to the same cortical layers in control samples, C) cellular aggregates added to specific cortical layers in case samples vs striated tissue structures added to the same cortical layers in control samples.

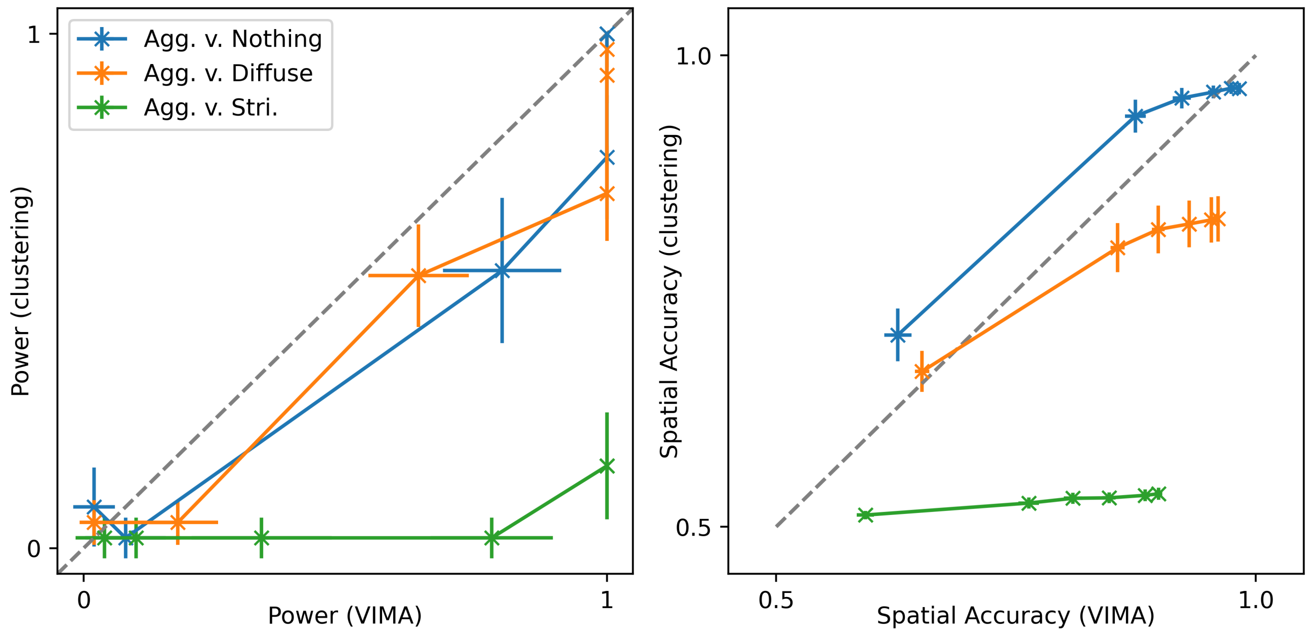

Supplementary Figure 3: Comparison of microniche- vs cluster-based association testing using VIMA's patch fingerprints. For each of the three signal types (aggregates versus nothing, aggregates versus diffuse infiltrate, aggregates versus striated structures), and each of 6 increasing noise levels, we show the power (left) and accuracy (right) of VIMA (x-axis) versus a cluster-based association test run using VIMA's patch fingerprints (y-axis).

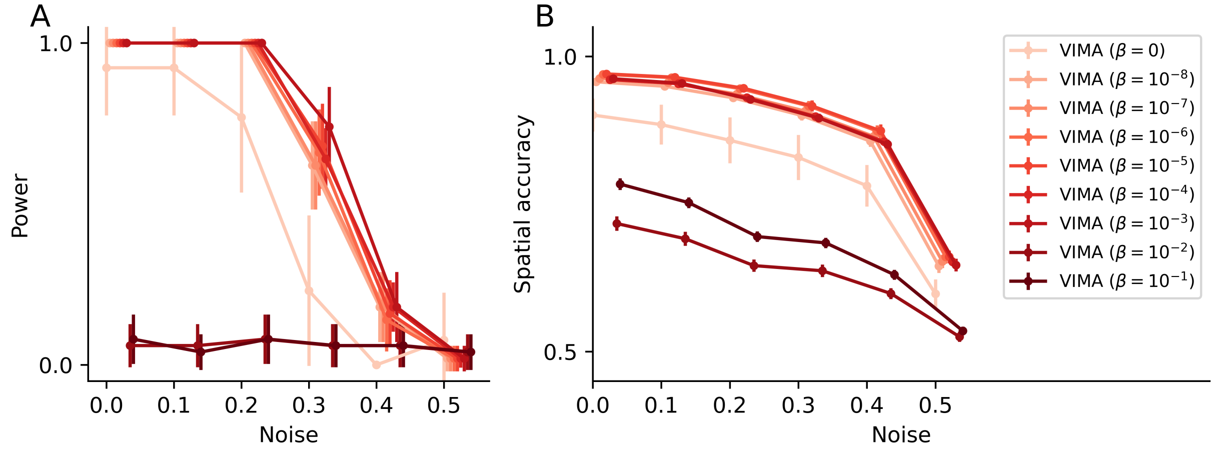

Supplementary Figure 4: Performance of VIMA with different amounts of variational regularization. For the cellular aggregates versus diffuse infiltrate signal, we varied the strength of the weight β given to the variational penalty in VIMA's VAE across orders of magnitude. Here we show the resulting A) power, and B) spatial accuracy as functions of noise, as in Figure 2.

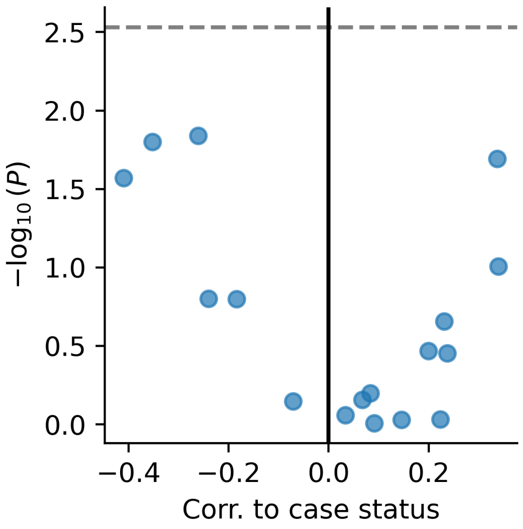

Supplementary Figure 5: Case-control analysis of Alzheimer's dataset with cell-type abundance-based representation of patches and cluster-based association testing. We represented each patch using its cell-type abundance profile, clustered the patches, and then tested each cluster for association and used a permutation-based approach analogous to VIMA's to determine P-values. In the plot, each dot represents a cluster and we plot the correlation of cluster abundance with case status against the permutation-based P-value for the association. The threshold for significance after Bonferroni correction for the number of clusters tested is shown as a dotted horizontal line.

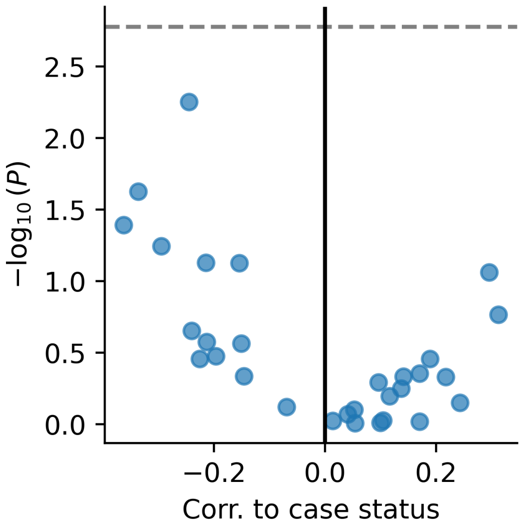

Supplementary Figure 6: Case-control analysis of Alzheimer's dataset with average expression-based representation of patches and cluster-based association testing. We represented each patch using its average expression profile, clustered the patches, and then tested each cluster for association and used a permutation-based approach analogous to VIMA's to determine P-values. In the plot, each dot represents a cluster and we plot the correlation of cluster abundance with case status against the permutation-based P-value for the association. The threshold for significance after Bonferroni correction for the number of clusters tested is shown as a dotted horizontal line.

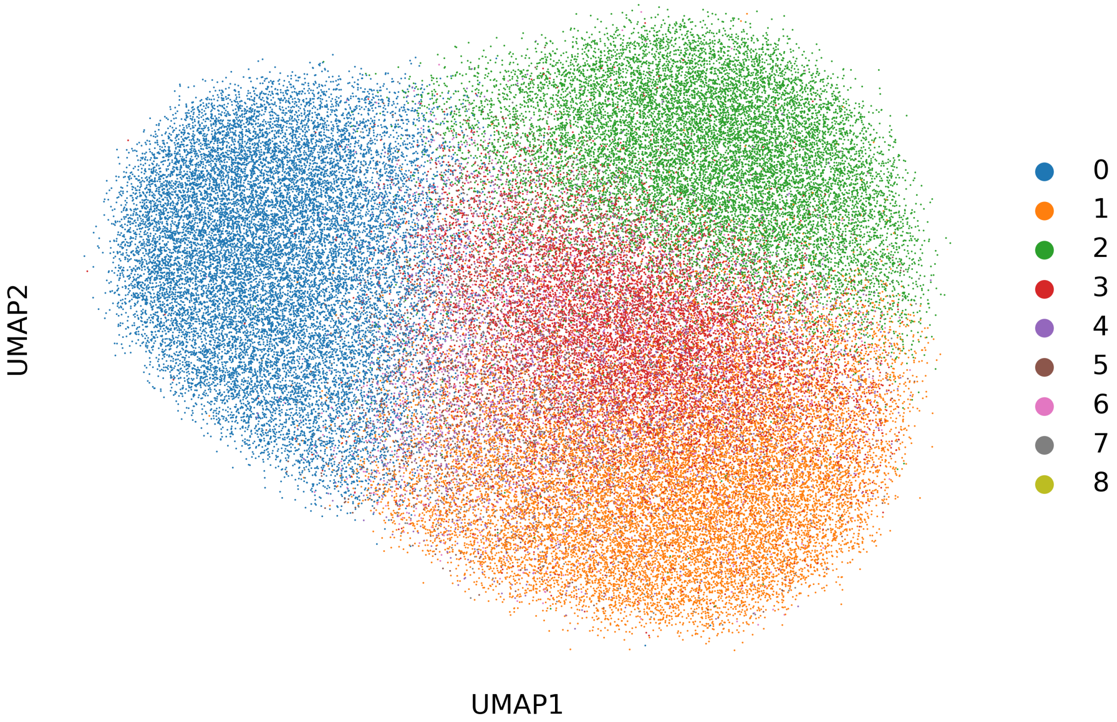

Supplementary Figure 7: Clustering of VIMA patch fingerprints in the Alzheimer's dataset. The UMAP visualizes the nearest-neighbor graph built using VIMA's patch fingerprints in the Alzheimer's dataset. We clustered the patches using Leiden clustering with default parameters and color each patch according to the cluster to which it belongs.

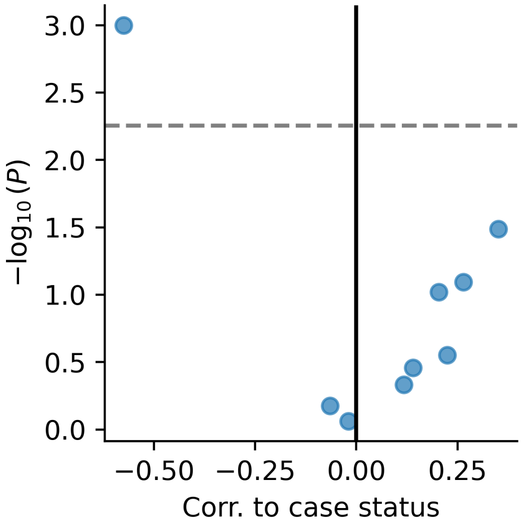

Supplementary Figure 8: Case-control analysis of Alzheimer's dataset with patch fingerprints used to represent patches and cluster-based association testing. We represented each patch using its patch fingerprint, clustered the patches, and then tested each cluster for association and used a permutation-based approach analogous to VIMA's to determine P-values. In the plot, each dot represents a cluster and we plot the correlation of cluster abundance with case status against the permutation-based P-value for the association. The threshold for significance after Bonferroni correction for the number of clusters tested is shown as a dotted horizontal line.

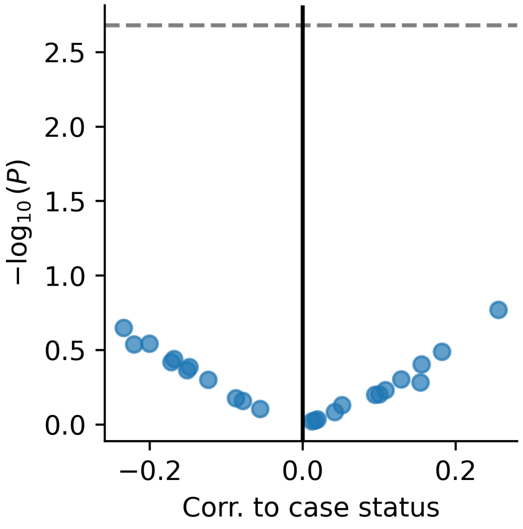

Supplementary Figure 9: Case-control analysis of Alzheimer's dataset using sample-wide cell-type composition to represent each sample. We represented each sample using its cell type abundance profile, and then tested each cell type for association and used a permutation-based approach analogous to VIMA's to determine P-values. In the plot, each dot represents a cell type and we plot the correlation of cell-type abundance with case status against the permutation-based P-value for the association. The threshold for significance after Bonferroni correction for the number of cell types tested is shown as a dotted horizontal line.

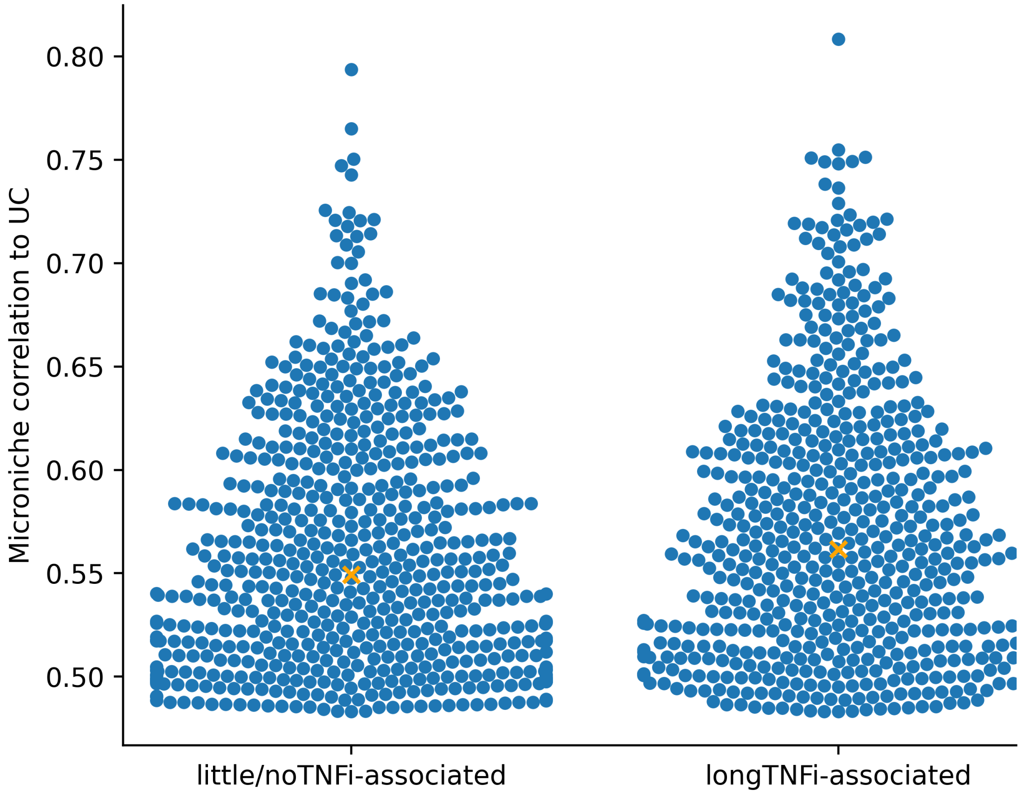

Supplementary Figure 10: Comparison of UC-vs-control signal to longTNFi-vs-little/noTNFi signal. For all the UC-associated microniches that had a significant correlation to longTNFi status, we plot the correlation between the microniche's abundance and UC status (y-axis) against whether the patch was little/noTNFi-associated (right) or longTNFi-associated (left).

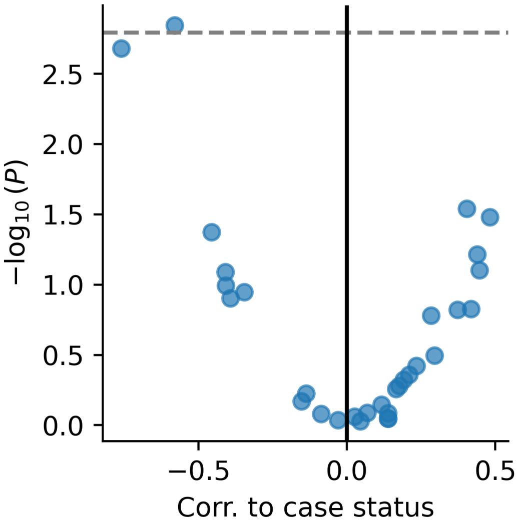

Supplementary Figure 11: Case-control analysis of UC dataset with average expression-based representation of patches and cluster-based association testing. We represented each patch using its average meta-marker profile, clustered the patches, and then tested each cluster for association and used a permutation-based approach analogous to VIMA's to determine P-values. In the plot, each dot represents a cluster and we plot the correlation of cluster abundance with case status against the permutation-based P-value for the association. The threshold for significance after Bonferroni correction for the number of clusters tested is shown as a dotted horizontal line.

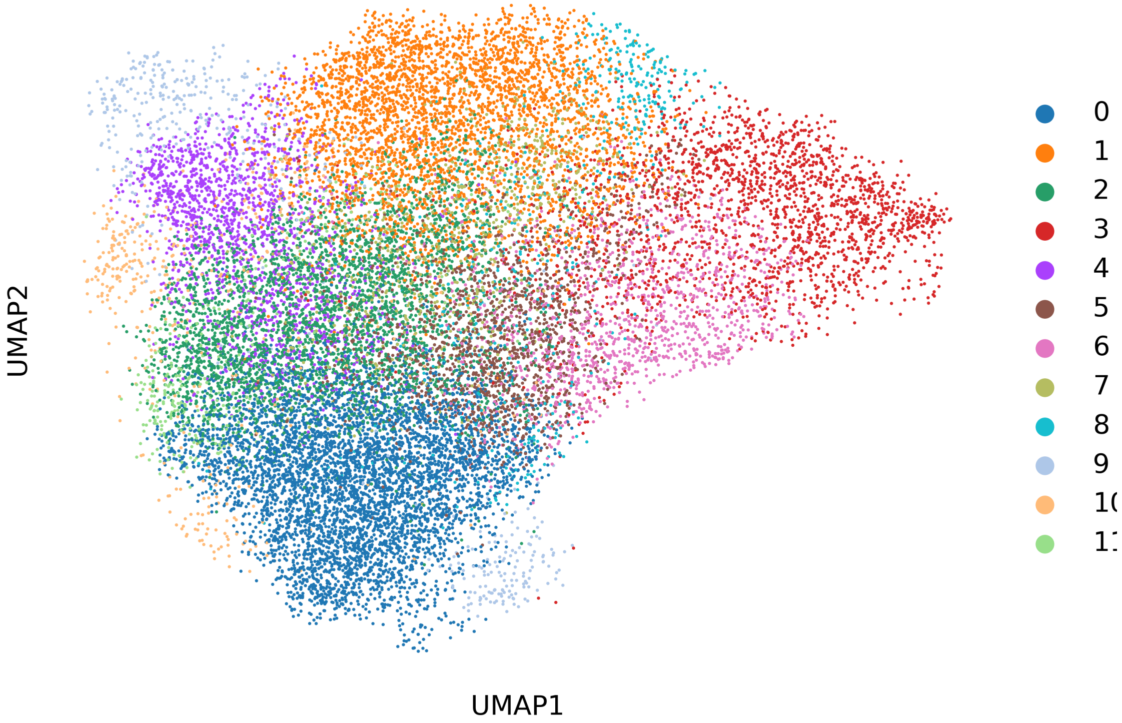

Supplementary Figure 12: Clustering of VIMA patch fingerprints in the UC dataset. The UMAP visualizes the nearest-neighbor graph built using VIMA's patch fingerprints in the UC dataset. We clustered the patches using Leiden clustering with default parameters and color each patch according to the cluster to which it belongs.

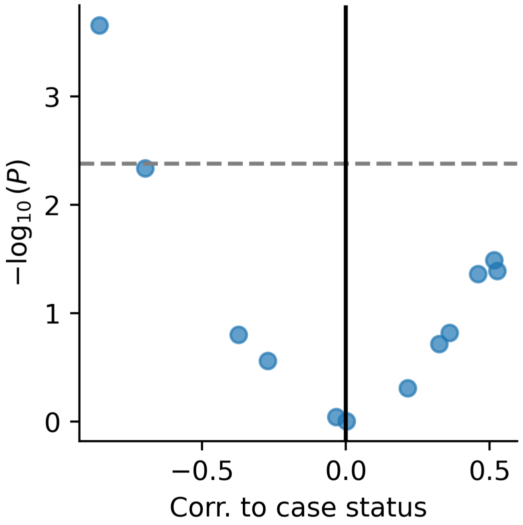

Supplementary Figure 13: Case-control analysis of UC dataset with patch fingerprints used to represent patches and cluster-based association testing. We clustered the patches in the UC dataset according to their VIMA fingerprints, and then tested each cluster for association and used a permutation-based approach analogous to VIMA's to determine P-values. In the plot, each dot represents a cluster and we plot the correlation of cluster abundance with case status against the permutation-based P-value for the association. The threshold for significance after Bonferroni correction for the number of clusters tested is shown as a dotted horizontal line.

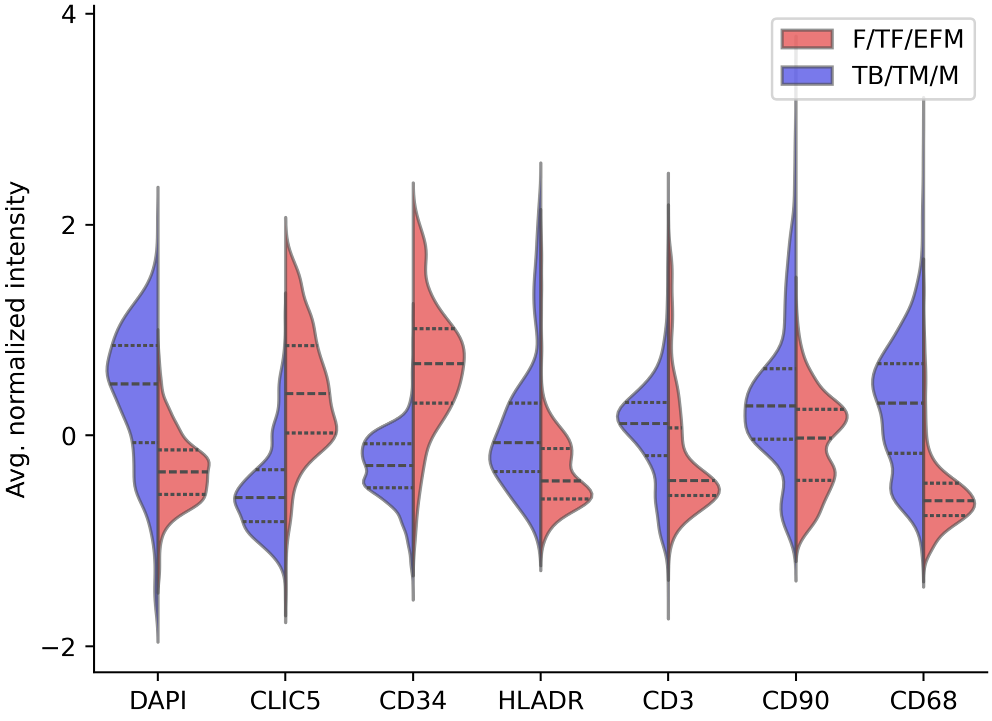

Supplementary Figure 14: Differentially abundant markers between fibroblast-rich-associated and fibroblast-poor-associated patches in the RA dataset. For each of the 7 markers in the dataset, we show the distribution of average marker intensity in patches anchoring microniches significantly associated with F/TF/EFM CTAPs versus those anchoring microniches significantly associated with M/TM/TB CTAPs.

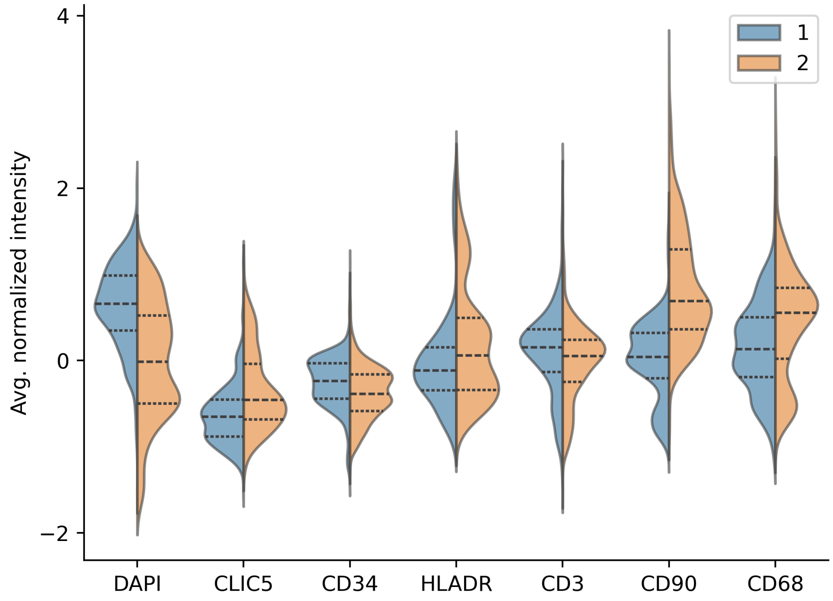

Supplementary Figure 15: Differentially abundant markers between the two clusters of fibroblast-poor-associated patches in the RA dataset. For each of the 7 markers in the dataset, we show the distribution of average marker intensity in patches assigned to cluster 1 from Figure 6 versus the average marker intensity in patches assigned to cluster 2 from Figure 6.

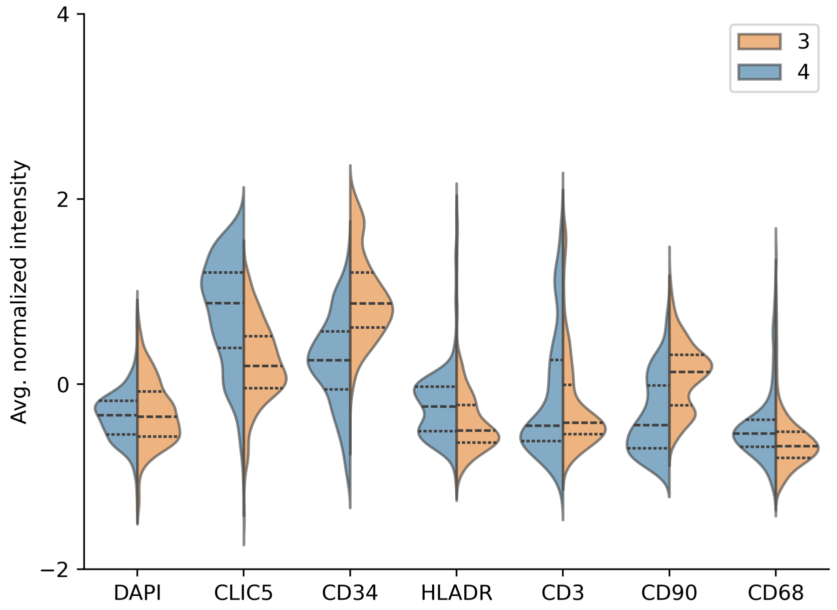

Supplementary Figure 16: Differentially abundant markers between the two clusters of fibroblast-rich-associated patches in the RA dataset. For each of the 7 markers in the dataset, we show the distribution of average marker intensity in patches assigned to cluster 3 from Figure 6 versus the average marker intensity in patches assigned to cluster 4 from Figure 6.

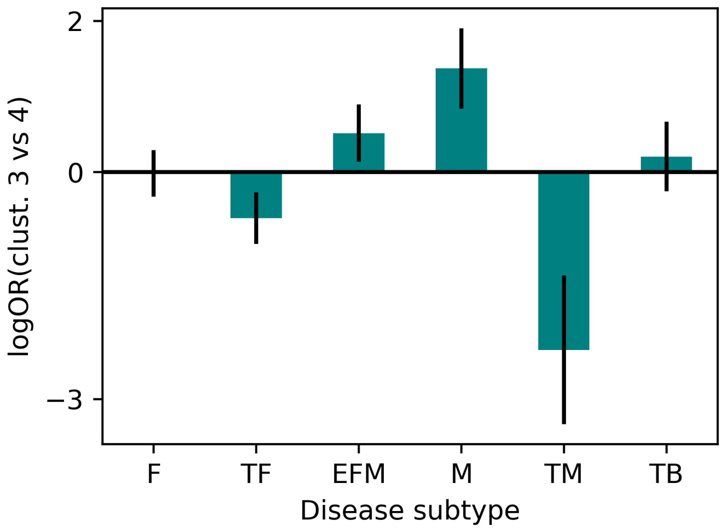

Supplementary Figure 17: Enrichment of cluster 3 vs cluster 4 among the full set of 6 disease subtypes in the RA dataset. For each CTAP (disease subtype), we show the log-odds-ratio for a randomly chosen patch from clusters 3 and 4 belonging to cluster 3 (positive log-odds) vs cluster 4 (negative log-odds), with 95% confidence intervals.

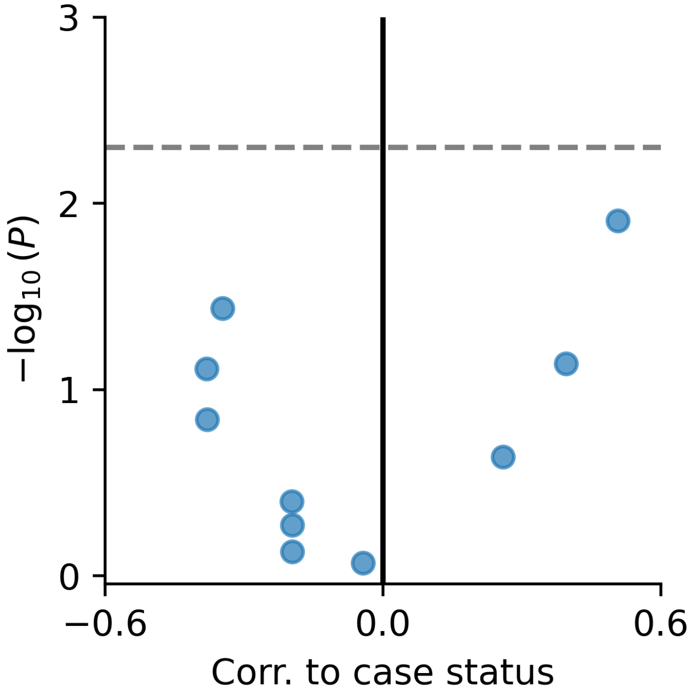

Supplementary Figure 18: Case-control analysis of RA dataset with average expression-based representation of patches and cluster-based association testing. We represented each patch using its average meta-marker profile, clustered the patches, and then tested each cluster for association and used a permutation-based approach analogous to VIMA's to determine P-values. In the plot, each dot represents a cluster and we plot the correlation of cluster abundance with case status against the permutation-based P-value for the association. The threshold for significance after Bonferroni correction for the number of clusters tested is shown as a dotted horizontal line.

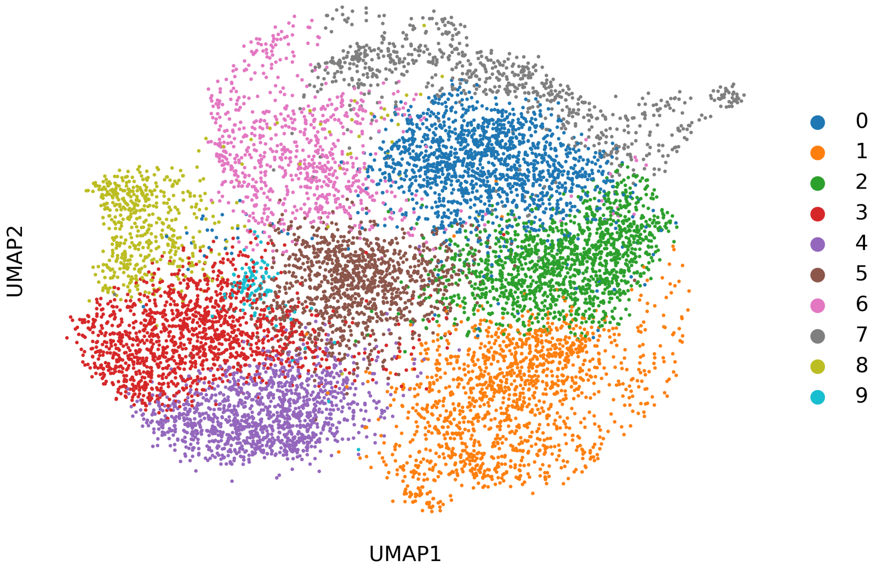

Supplementary Figure 19: Clustering of VIMA patch fingerprints in the RA dataset. The UMAP visualizes the nearest-neighbor graph built using VIMA's patch fingerprints in the RA dataset. We clustered the patches using Leiden clustering with default parameters and color each patch according to the cluster to which it belongs.

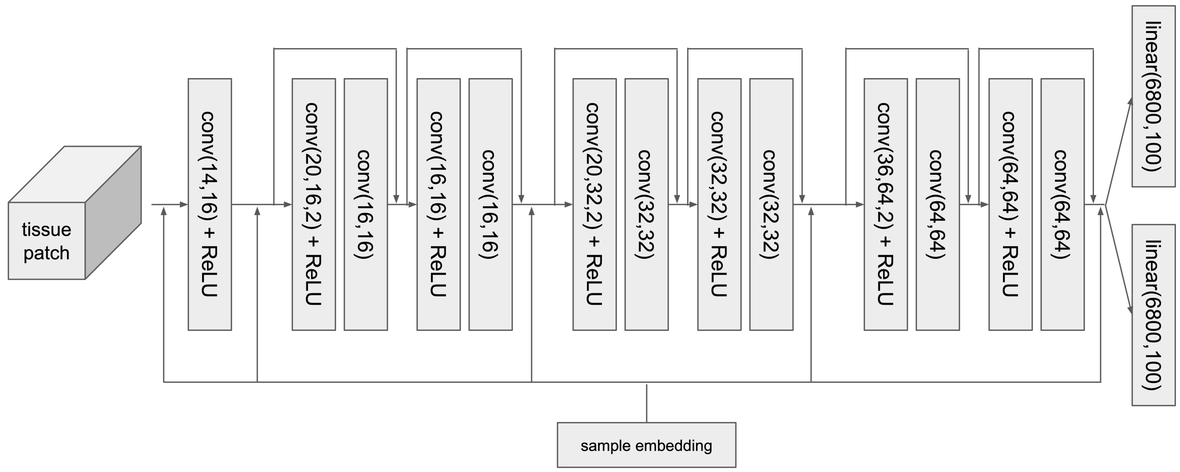

Supplementary Figure 20: Schematic depiction of the architecture of the VIMA encoder. The encoder takes as input a tissue patch that by default is of size 40 pixels by 40 pixels by 10 meta-markers. This is passsed through a series of convolutional layers; we denote each convolution by specifying first the number of color channels that it takes as input, second the number of color channels it produces as output, and finally the stride (if it differs from 1). The sample embedding is a 4-dimensional vector that is unique to each sample and learned along with the weights of the model. Each arrow emanating from the sample embedding represents concatenation of these 4 numbers as 4 additional spatially homogeneous color channels. The arrows that begin before and end after some of the convolutional layers represent simple addition, with a convolutional layer to reduce the x- and y- dimensions of the patch if necessary; these “skip” connections, which allow the convolutional layers to learn residual rather than cumulative signal, are the reason for the “Res” in the name “ResNet”.
